# Nasopharyngeal carcinoma: current views on the tumor microenvironment's impact on drug resistance and clinical outcomes

**DOI:** 10.1186/s12943-023-01928-2

**Published:** 2024-01-22

**Authors:** Huai Liu, Ling Tang, Yanxian Li, Wenji Xie, Ling Zhang, Hailin Tang, Tengfei Xiao, Hongmin Yang, Wangning Gu, Hui Wang, Pan Chen

**Affiliations:** 1 Hunan Cancer Hospital and the Affiliated Cancer Hospital of Xiangya School of Medicine, Central South University, Changsha, 410013 Hunan China; 2 State Key Laboratory of Oncology in South China, Guangdong Provincial Clinical Research Center for Cancer, Sun Yat-Sen University Cancer Center, Guangzhou, China

**Keywords:** Nasopharyngeal carcinoma, Tumor microenvironment, Immune escape, Drug resistance

## Abstract

The incidence of nasopharyngeal carcinoma (NPC) exhibits significant variations across different ethnic groups and geographical regions, with Southeast Asia and North Africa being endemic areas. Of note, Epstein-Barr virus (EBV) infection is closely associated with almost all of the undifferentiated NPC cases. Over the past three decades, radiation therapy and chemotherapy have formed the cornerstone of NPC treatment. However, recent advancements in immunotherapy have introduced a range of promising approaches for managing NPC. In light of these developments, it has become evident that a deeper understanding of the tumor microenvironment (TME) is crucial. The TME serves a dual function, acting as a promoter of tumorigenesis while also orchestrating immunosuppression, thereby facilitating cancer progression and enabling immune evasion. Consequently, a comprehensive comprehension of the TME and its intricate involvement in the initiation, progression, and metastasis of NPC is imperative for the development of effective anticancer drugs. Moreover, given the complexity of TME and the inter-patient heterogeneity, personalized treatment should be designed to maximize therapeutic efficacy and circumvent drug resistance. This review aims to provide an in-depth exploration of the TME within the context of EBV-induced NPC, with a particular emphasis on its pivotal role in regulating intercellular communication and shaping treatment responses. Additionally, the review offers a concise summary of drug resistance mechanisms and potential strategies for their reversal, specifically in relation to chemoradiation therapy, targeted therapy, and immunotherapy. Furthermore, recent advances in clinical trials pertaining to NPC are also discussed.

## Background

Nasopharyngeal carcinoma (NPC) is a form of head and neck cancer originating from the epithelium of the nasopharynx. According to data from the US National Cancer Institute, NPC is diagnosed in fewer than one individual per 100,000 worldwide annually. However, NPC is endemic to specific geographical regions, such as Southeast Asia, North Africa, and the Arctic [1], where high incidence has been reported. The prevalence is higher in males than in females in both high- and low-incidence areas, with a male-to female ratio of approximately 3:1. Currently, NPC is classified into keratinizing carcinoma/squamous cell carcinoma, non-keratinizing carcinoma, with differentiated and undifferentiated variants, and basaloid squamous cell carcinoma. The keratinizing subtype accounts for less than 20% of all cases in the United States while the nonkeratinizing subtype represents the endemic form of NPC and is found mostly in Asia.

The etiology of NPC is multifactorial and complex. Exposures to environmental carcinogens, and genetic predisposition as demonstrated by the geographic distribution and overwhelming incidence in the Chinese population [2]. Epstein-Barr virus (EBV) infection, which accounts for most of the cases in all subtypes of non-keratinizing NPC [3]. It has also been speculated that co-infection with other viruses, such as human papillomavirus (HPV) and human herpesvirus type 6 (HHV-6), may also play a role in the development of NPC. In addition, causal factors, including tobacco, alcohol, and nitrosamine-containing food consumption, have a synergistic effect with EBV infection on the risk of NPC development [4].

Depending on the staging, NPC patients will receive single treatment or combination treatment of radiotherapy, chemotherapy, targeted therapy, or immunotherapy. Surgical options are limited due to the deep tumor localization and complex anatomical structure of the tumor site, but still provide benefit to recurrent NPC [5]. Factors that contribute to NPC treatment response include the stage of the cancer, the therapeutic approaches, the treatment-related side effects, and the occurrence of drug resistance. Risk factors associated with NPC are listed in Table 1. In recent decades, tumor microenvironment (TME) is being recognized to play a significant role in promoting tumor progression, metastasis, and mediating drug resistance. The TME of NPC is a complicated network between tumor-associated cells and noncellular components, together shaping an environment that favors tumor development. In this review, we summarize the NPC therapeutic options, describe the role of TME in the development of NPC, and specifically discuss the drug resistance mechanisms as well as reversal strategies.

**Table 1:** Risk factors associated with NPC

Risk factors	OR (95% CI)	References
EBV infection	8.69 (5.79–13.03)	[6]
Malarial infection	2.2 (N/A)	[7]
Smoking habit	1.34 (1.15–1.57)	[8]
Salt-preserved food	1.36 (1.27–1.46)	[9]
Plant-based diet	0.48 (0.38–0.59)	[10]
Alcohol consumption	1.41 (1.27–1.57)	[9]
Animal-based diet	2.26 (1.86–2.77)	[10]
Exposure to wood dust	5.82 (2.50–13.6)	[11]
Exposure to formaldehyde	6.79 (2.21–15.85)	[12]
NPC family history	3.09 (1.97–4.86)	[11]
CYP2E1 RsaI/PstI polymorphism	2.72 (1.73–4.25)	[13]
GSTM1 polymorphism	1.53 (1.35–1.74)	[14]
ALDH2 rs671 polymorphism	1.23 (1.03–1.48)	[15]
p53 Arg72Pro SNP	1.28 (1.17–1.40)	[16]
MMP-2 1306C > T SNP	2.22 (0.50–9.91)	[17]

*Abbreviations*: *OR* odd ratio, *CI* confidence interval, *SNP* single nucleotide polymorphism

## NPC therapeutic approaches

### Radiotherapy

NPC has been treated with radiotherapy (RT) for over 70 years because it is a radiosensitive tumor and the anatomic location limits surgery. NPC is highly sensitive to ionizing radiation (IR), and therefore RT is the mainstay option and the backbone of treatment for all stages of NPC without distant metastases. IR can inhibit tumor progression by indirectly inducing DNA damage through direct ionization or by stimulating reactive oxygen species (ROS) production [18]. With the development of photon RT in the past few decades, the treatment strategies have progressed from conventional two-dimensional RT to three-dimensional conformal RT (3D-CRT), as well as the recent breakthrough in intensity-modulated RT (IMRT). Currently, IMRT represents the most widely selected option that provides substantial locoregional control and overall survival rate (OS) and reduced toxicities compared to 2D- or 3D-RT [19]. For patients with early-stage NPC, the standard treatment is RT monotherapy, and the prognosis is desirable. A Chinese clinical study reported the treatment outcomes of patients with early-stage NPC after RT monotherapy [20]. The 5-year OS rate for the whole sample size (*n* = 362) was 85%. In addition, the 5-year local recurrence-free survival and 5-year regional recurrence-free survival rates had no significant difference between different T and N stages on prognosis. In contrast, patients with locally advanced NPC have worse therapeutic outcomes and are usually treated with combined chemoradiotherapy. Fang et al. conducted a longitudinal study to investigate the survival outcomes for NPC patients treated with 3D-CRT and IMRT [21]. Both therapies showed remarkable effect, with more than 93% 3-year OS, towards early stages NPC, but the OS rates reduced to 70% in stage IV. Furthermore, RT alone demonstrated 100% 3-year metastasis-free survival rate in stage I-IIa, but only achieved 60% response in stage IV. The major limitation of RT lies in the insufficient local control for advanced stages tumor, severe late adverse events, and the lack of adaptive re-planning during RT course [22].

### Chemotherapy

Chemotherapy has been evaluated as part of the combination with RT to reduce tumor load and eradicate micro-metastases. Common chemotherapeutic agents used in NPC treatment are platinum-based drugs, paclitaxel, gemcitabine, and 5-fluorouracil (5-FU). Multiple clinical trials have tested the effect of induction, concomitant, and adjuvant chemotherapy [23, 24]. These trials were conducted with different drug combinations and dosing strategies. Nonetheless, the outcomes were discouraging and sparked a contentious discussion regarding the utilization of chemotherapy for both local control and distant metastases. Monotherapy of methotrexate, 5-FU, cisplatin, and carboplatin achieved response rate of 15% to 31% [19]. Neoadjuvant chemotherapies for NPC are cisplatin-based treatments plus gemcitabine, etoposide, or 5-FU, aiming to reduce tumor volume prior to radiotherapy and to eradicate microscopic metastasis [25]. The mainstream adjuvant chemotherapy regime for locally advanced NPC is the combination of cisplatin with 5-FU. Although neoadjuvant and adjuvant chemotherapy has reported encouraging response rates in treating locoregionally advanced NPC, so far none of the trials has been able to demonstrate an OS benefit [26]. A retrospective analysis by Song et al. described that neoadjuvant chemotherapy offered no additional benefit to treatment with RT alone. Instead, introducing chemotherapy may cause deleterious effect on stage II NPC by delaying RT treatment [27]. Such findings are supported by a meta-analysis that included 8 trials with 1753 patients, but the study also suggested an OS benefit with concomitant chemotherapy [28]. Recently, Wang et al. conducted a meta-analysis of 8 studies with 2605 patients bearing locally advanced NPC [29]. The result suggested that induction chemotherapy plus RT was as effective as induction chemotherapy plus concurrent chemoradiotherapy. Moreover, the exclusion of concurrent chemotherapy reduced the incidence of treatment-related acute hematological toxicity. Therefore, the most promising regimen could be concurrent chemoradiotherapy for patients with locoregionally advanced diseases. Importantly, the outcome of chemotherapy can be affected by NPC stages, drug combinations, and time sequences.

### Targeted therapy

**Fig. 1:**
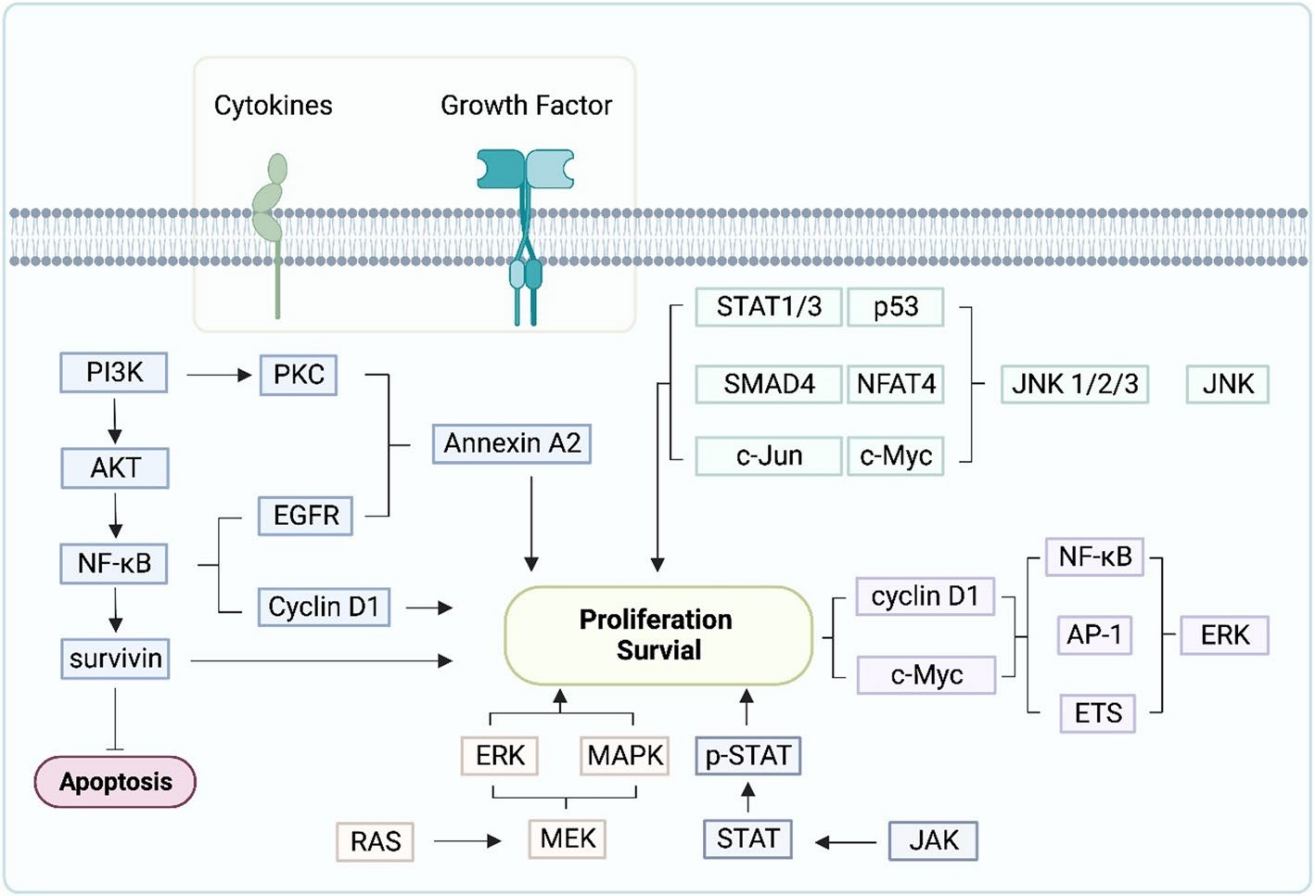
Signaling pathways contributed to the progression of nasopharyngeal carcinoma. Initiation of the cell signaling begins with cytokines and growth factors stimulation. The PI3K pathway leads to apoptosis inhibition and tumor cell proliferation. Other signaling pathways including JNK, ERK, JAK, and RAS promote tumor cell proliferation

NPC cells can utilize several signaling pathways to promote cell proliferation and inhibit apoptosis, as shown in Fig. 1. Therefore, targeting these signaling proteins can be an effective strategy in NPC. Hyperactivation of PI3K-AKT signaling cascade is one of the crucial mechanisms in NPC cell proliferation and survival. EBV-encoded latent membrane protein 1 (LMP1) also relies on PI3K-AKT pathway to mediate fibroblast transformation [30]. Alteration of PI3K gene such as gene amplification, mutations have been correlated with aggressive tumor behavior and poor survival in NPC patients [31]. A nonselective PI3K inhibitor LY294002 was reported to induce cell apoptosis by inhibiting the activation of PI3K-AKT signaling [32]. Mitogen-activated protein kinase (MAPK) signaling pathway regulates a wide range of cellular processes, such as cell proliferation, differentiation, migration, and apoptosis [33]. It consists of three pathways including extracellular-signal-regulated kinase 1 and 2 (ERK1/2), c-Jun N-terminal kinase 1, 2, and 3 (JNK1/2/3), and p38 MAPK. MAPKs are activated upon ligand binding to receptors such as EGFR, FGFR, VEGFR, then transduced through RAS signaling and leads to phosphorylation of MAPK substrates, leading to regulation of target gene activity [34]. The activation of MAPK pathway has been shown to promote NPC tumor development [35, 36], angiogenesis [37], metastasis [38, 39], and the formation of inflammatory TME [40]. As shown in Fig. 1, NF-κB is involved in multiple signaling pathways, representing a key multipotent transcription factor [41]. Studies have shown that EBV-encoded LMP1 could activate NF-κB to promote tumor cell metabolic reprogramming and modulate the immunosuppressive TME [42]. While these signaling pathways are critical to NPC tumor progression, the clinical efficacy of therapeutic inhibitors requires further evaluation.

Small molecule inhibitors targeting EGFR and VEGFR are considered as second line therapy for recurrent or metastatic (R/M) NPC patients. Evidence suggests that VEGFR-2 is highly expressed in NPC, which correlated with worse progression-free survival (PFS) [43]. The activation of VEGF signaling pathway promotes tumor growth, metastasis, and angiogenesis, therefore targeting VEGFR could provide benefit for NPC patients. Sorafenib, sunitinib, famitinib, and pazopanib are multi-targeted tyrosine kinase inhibitors of VEGFR. The efficacy of sorafenib was evaluated in R/M NPC patients as monotherapy or in combination with chemotherapy [44, 45]. Sorafenib was shown to be clinically effective and fairly well tolerated in patients with R/M NPC. Notably, sorafenib combined with cisplatin and 5-FU achieved promising objective response rate of 77.8% and median OS of 11.8 months. Clinical trials on sunitinib, pazopanib, and famitinib also suggest that these VEGFR inhibitors have therapeutic advantages in heavily chemotherapy-pretreated patients with R/M NPC. Recently, highly selective VEGFR-2 inhibitors anlotinib and apatinib have been investigated in R/M NPC. In a clinical trial with 33 enrolled patients (NCT03130270) who experienced disease progression following chemotherapy, apatinib monotherapy exhibited notable therapeutic effectiveness and a well-tolerated safety profile [46]. Anlotinib is undergoing multiple clinical trials to evaluate its efficacy, either as a standalone treatment (NCT03906058) or in combination with chemoradiation (NCT05232552) for patients with NPC. The clinical trials of small molecule inhibitors are summarized in Table 2.

**Table 2:** Small molecule inhibitors under clinical investigation

Target	Agent	The National Clinical Trial number	Year of completion
**EGFR, VEGFR, FGFR**	Apatinib	NCT02874651	Completed, 2020–08
		NCT04586088	2023–09
		NCT04350190	Completed, 2022–09
		NCT03180476	Completed, 2020–04
		NCT05549466	2025–09
	Anlotinib	NCT03639467	2023–08
		NCT05232552	2024–12
		NCT03906058	Completed, 2022–04
		NCT05981157	2025–12
		NCT05807880	2025–10
	Surufatinib	NCT04955886	2023–08
	Sorafenib	NCT02035527	Completed, 2017–04
	Donafenib	NCT02698111	Terminated, 2021–10
**PI3K**	IPI-549	NCT03795610	2023–06
	BKM120	NCT02113878	Completed, 2022–01
		NCT01816984	Completed, 2020–09
**PARP**	Fluzoparib	NCT04978012	2025–12
	Niraparib	NCT05162872	2023–10
**HDAC**	Nanatinostat	NCT05166577	2025–10
**mTOR**	RAPA-201	NCT05144698	2024–12
**CDK4/6**	Dalpiciclib	NCT05724355	2024–10
**EBNA1**	VK-2019	NCT04925544	2026–02

Clinical investigations have confirmed elevated expression of EGFR as well as EGFR gene amplification in non-keratinizing NPC tumors, a correlation that has been linked to unfavorable treatment outcomes [21]. Activation of EGFR results in hyperactivity of the downstream signaling cascades such as RAS/ERK pathways, leading to uncontrolled cell proliferation [36]. These observations provided a rationale for the exploration of EGFR inhibitors in recurrent or metastatic (R/M) NPC. Multiple clinical trials were undertaken to assess the effectiveness of EGFR inhibitors, such as gefitinib and erlotinib, in patients with R/M NPC who had previously undergone chemotherapy [47–49]. Although both inhibitors showed favorable safety profile, they failed to demonstrate meaningful clinical and survival benefits for R/M NPC patients. Recently, another EGFR inhibitor icotinib was evaluated in combination with IMRT in patients with local NPC. While the combination was shown to be well tolerated, the efficacy needs further investigation [50].

In summary, the use of small molecule inhibitors in the context of recurrent or metastatic (R/M) NPC did not yield significant clinical benefits, this could be partially attributed to the absence of reliable biomarkers, a lack of robust clinical trials, and the short duration of follow up studies. Further studies are warranted to discover better therapeutic options and establish a definitive role for targeted therapy in the management of R/M NPC.

### Immunotherapy

The immune landscape of NPC is generally described as a highly immune inflammatory environment, with a close association with EBV infection, high PD-L1 level, and lymphocytic infiltration [51]. Multiple immunotherapies for NPC are currently under investigation, which include cancer vaccines, immune checkpoint inhibitors (ICIs), and adoptive cell therapy (ACT). To date, immunotherapy for NPC has demonstrated significant benefits in multiple clinical trials, offering novel avenues to improve the therapeutic responses among patients with NPC. A summary of immunotherapy clinical trials is presented in Table 3.

**Table 3:** Immunotherapy clinical trials within the last 10 years

Target	Treatment	The National Clinical Trial number	Year of completion
**Immune cells**	T cell	NCT03044743	2022–03
		NCT04509726	2023–08
		NCT05592626	2026–10
	Nature killer cell	NCT03007836	Completed, 2019–06
	Dendritic cell	NCT04476641	2022–12
**Anti-PD1**	Toripalimab	NCT03925090	2023–10
		NCT04446663	2024–12
		NCT04447326	2026–06
		NCT04517214	2026–12
		NCT05147844	2024–03
		NCT05229315	2023–12
		NCT05385926	2024–04
		NCT05484375	2029–09
		NCT05813626	2027–10
		NCT04890522	2028–12
		NCT03581786	2022–10
		NCT04453813	2027–07
		NCT04778956	2033–03
		NCT05340491	2027–12
		NCT05955105	2026–07
		NCT03930498	2025–12
	Nivolumab	NCT02339558	Completed, 2018–06
		NCT03267498	2024–04
		NCT03097939	2024–12
		NCT04875611	2025–12
		NCT05904080	2028–06
		NCT04910347	2025–12
		NCT06019130	2028–01
		NCT04458909	Terminated, 2023–08
		NCT06029270	2029–04
	Pembrolizumab	NCT03734809	2024–12
		NCT03813394	2024–03
		NCT03082534	2024–05
		NCT03809624	2025–12
		NCT04825990	2028–03
		NCT02611960	Completed, 2022–09
		NCT03674567	2023–12
		NCT02538510	Completed, 2023–09
	Sintilimab	NCT04872582	2024–10
		NCT04917770	2024–06
		NCT03700476	2025–01
		NCT03619824	2024–03
		NCT05201859	2026–02
		NCT05417139	2025–07
	Camrelizumab	NCT04221516	2024–02
		NCT04782765	2025–03
		NCT05011227	2025–08
		NCT05097209	2026–04
		NCT05128201	2026–05
		NCT03427827	2026–02
		NCT03707509	2022–12
		NCT04453826	2028–09
		NCT04944914	2026–06
		NCT05524168	2025–09
	Penpulimab	NCT03866967	2023–12
		NCT04220307	Completed, 2022–08
		NCT04974398	2026–09
	Tislelizumab	NCT04833257	2026–11
		NCT04870905	2026–05
		NCT05448885	2025–12
		NCT05211232	2027–03
		NCT03924986	2024–06
	Tirelizumab	NCT05092217	2024–10
	Peramprizumab	NCT05193617	2027–01
	Triprilimab	NCT04421469	2023–06
**PD-1/CTLA-4**	Cadonilimab	NCT05790200	2025–09
		NCT05587374	2027–01
**PD-L1**	Envafolimab	NCT05397769	2026–12
	Durvalumab	NCT04447612	2024–12
	KL-A167	NCT05294172	2025–05
**VEGF**	Bevacizumab	NCT05341193	2025–12
		NCT05898256NCT03074513	2026–07
		NCT05063552	2024–06
			2027–12
**EGFR**	Nimotuzumab	NCT03708822	2026–12
		NCT04223024	2026–12
		NCT04456322	2025–06
**TIM-3**	TQB2618	NCT05563480	2024–05
**HGF**	Ficlatuzumab	NCT03422536	Completed, 2022–04
**TGF-β receptor**	Bintrafusp Alfa	NCT04396886	2022–12

#### Targeting EBV

Since EBV is present in virtually all poorly differentiated and undifferentiated non-keratinizing NPC tumor cells but rarely in normal cells, it represents a very specific diagnostic biomarker and an attractive therapeutic target. Numerous studies have demonstrated that EBV can induce genetic instability premalignant cells, promote tumor development through shaping an immunosuppressing TME, and facilitate tumor metastasis and invasion by inducing EMT. Given the oncogenic role of EBV in NPC, multiple clinical trials are initiated to explore the therapeutic value of targeting EBV [52]. In order to target EBV, peptide vaccine and viral vaccine, have been investigated in the context of NPC treatment [53]. Prophylactic EBV-vaccine aims to restrict primary infection and reduce the risk of EBV-associated diseases. To this end, vaccines targeting different EBV antigens have been developed and are under clinical investigation (NCT01094405, NCT01800071). The lytic antigen gp350 is an ideal candidate given its critical role in guiding primary B-cell infection and neonatal infection [54]. Therefore, vaccines against EBV gp350 are expected to prevent primary infection. Notably, the NIH recently announced an early-stage clinical trial to evaluate the safety and immune response of an EBV gp350-Ferritin nanoparticle vaccine. Another type of vaccine targets the latent life cycle antigens such as the EBV nuclear antigens (EBNAs) and latent membrane proteins (LMPs). This approach may be adapted to eradicate latently-infected reservoirs. Clinical trials have been conducted to evaluate the vaccines against two main EBV proteins, EBNA1 and LMP2 [55, 56]. For instance, a phase I clinical trial (NCT01256853) tested a recombinant vaccinia virus encoding an EBNA1/LMP2 fusion protein to boost T cell immunity [57]. The data show that the vaccine can induce a dose dependent EBNA1/LMP2 response with a safe profile, supporting the initiation of a phase II trial.

#### Adopted cell therapy

In addition to cancer vaccines, directing attention towards EBV through Adoptive Cell Therapy (ACT) has emerged as a new option with potential for superior effectiveness and a favorable safety profile. The potential of ACT was first noted by Smith et al., who suggested that it could potentially prevent tumor development and extend patient survival [58]. Targeting EBV-specific antigens LMP1 and EBNA1 with cytotoxic T lymphocytes (CTLs) represents one of the most promising immunotherapies for NPC. LMP1 and EBNA1 are highly immunogenic and successful targeting of EBV-specific antigens with CTLs may have long-lasting effects. Once activated, CTLs can continue to surveil the body for reappearance of EBV-infected cells, reducing the risk of recurrence. CTL-based therapies have fewer severe side effects compared to traditional chemotherapy or radiation therapy.

Other immune cells include dendritic cells (DC) which are potent antigen-presenting cells that play a central role in initiating and regulating anti-viral immune responses. Through genetic engineering DCs can present tumor antigens to naïve CD8^+^ T cells and turn them into tumor specific CTLs [59]. Currently, there are several clinical trials investigating the possibility of ACT against NPC and reported results demonstrated clinical benefits. A recent study by Li et al. reported that ACT is well tolerated following chemoradiotherapy in advanced NPC patients. More importantly, 19 of 20 patients exhibited an objective antitumor response, and 18 patients displayed disease-free survival more than 12 months after ACT, suggesting a sustained antitumor activity achieved by stimulating anti-EBV immune responses [60]. These observations align with results obtained from other clinical trials, including studies involving the combination of ACT with chemotherapy [35] and treatment in patients who did not respond to conventional therapies [36]. Furthermore, a systematic review conducted by Farooqi et al. reached a similar conclusion. In their analysis of 7 phase I/II clinical trials, they found minimal grade ≥ 3 adverse events, with the majority of such events being associated with chemotherapy [37]. Therefore, the combination of adoptive immunotherapy with traditional therapeutic approaches may achieve substantial treatment response while preserving a good safety profile.

#### Immune checkpoint inhibitors

ICIs represent a major class of immunotherapy across many cancers. Accumulating data has suggested the great potential of ICIs in the treatment of NPC. Most of the research are focusing on the development of programmed death-1/programmed death ligand-1 (PD-1/PD-L1) and cytotoxic T lymphocyte-associated protein 4 (CTLA-4) inhibitors. Tumor cells have the capacity to engage CTLA-4, thereby suppressing the initiation of T-cell responses and evading immune surveillance. Preclinical investigations have demonstrated that the inhibition of CTLA-4 can restore T-cell functions and facilitate the elimination of tumors. Ipilimumab and tremelimumab have been approved for adjuvant therapy of melanoma [61]. Ipilimumab is currently under phase 2 clinical trials for metastatic, recurrent, or last-staged NPC.CTLA-4 inhibitors are also under clinical investigation in other cancers, including lung cancer, prostate cancer, and NPC [62]. Cadonilimab is a bispecific antibody targeting both PD-1 and CTLA-4. The early phase clinical studies showed that cadonilimab achieved promising antitumor efficacy with a favorable safety profile, leading to its approval for recurrent or metastatic cervical cancer in China. It is now in multiple phase 2/3 clinical trials to investigate the antitumor efficacy in NPC [63]. PD-1 is an immune checkpoint found on T lymphocytes, whereas PD-L1 is present on both tumor cells and immune cells. Zhang and colleagues conducted an analysis of biopsies from 139 patients to assess the relationship between the expression levels of PD-1 and PD-L1 and treatment outcomes. Strikingly, PD-L1 which was identified in 95% of the patients was associated with an unfavorable prognosis. Co-expression of PD-1 and PD-L1 was associated with the poorest prognosis of disease-free survival. This finding agrees with studies in other cancers [64, 65]. Given that PD-1 and PD-L1 play critical roles in tumor survival and immune escape, treatment of NPC with ICIs has become a high interest research field. Many PD-1/PD-L1 inhibitors have advanced from preclinical studies to clinical trials for the treatment of recurrence/metastatic (R/M) NPC, including nivolumab, pembrolizumab, camrelizumab, toripalimab, tislelizumab [51]. The promising data have granted several approvals of PD-1 inhibitors for R/M NPC in combination with chemotherapy. A multinational study of nivolumab monotherapy in 44 patients demonstrated an overall response rate of 20.5%. In addition, the 1-year OS was 59% and 1-year PFS was 19.3%, suggesting that it has promising clinical activity in heavily pretreated R/M NPC. The study also showed that patients with PD-L1 positive tumors had higher response rate compared to those with PD-L1 negative tumors. In a phase 3 clinical study, toripalimab in combination with gemcitabine-cisplatin chemotherapy exhibited a significant improvement in median PFS (11.7 vs 8.0 months), and a 40% reduction in risk of death compared to placebo arm. The addition of toripalimab to chemotherapy had a similar incidence of grade ≥ 3 adverse events, with a more frequent immune-related adverse events (39.7%vs 18.9%) [66]. Notably, toripalimab is the first anti-PD-1 antibody that got approved for R/M NPC in China, representing a breakthrough of immunotherapy in NPC treatment [66]. Tislelizumab was approved by the China National Medical Products Administration for R/M NPC in 2022, based on the significant efficacy in phase 3 RATIONALE-309 trial.

## Components of the NPC tumor microenvironment

**Fig. 2:**
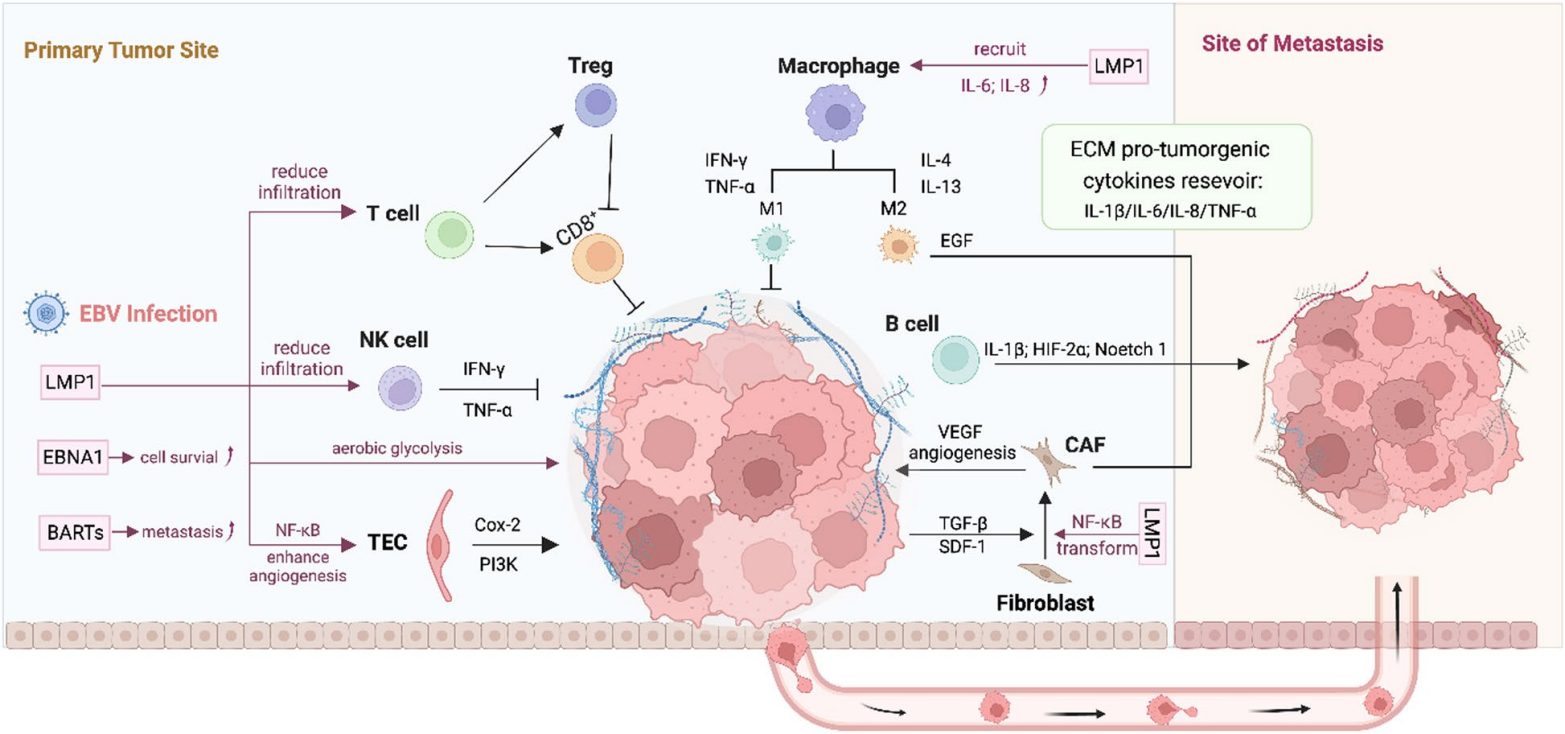
Interaction of NPC cells with the tumor microenvironment. EBV-encoded proteins promote tumor development by interacting with tumor-associated cells while limiting the immune infiltration of T cells and NK cells. Immune cells including nature killer (NK) cells, cytotoxic CD8^+^ T cells, M1 macrophage can inhibit NPC tumor growth. In contrast, regulatory T cells (Treg), M2 macrophage, and B cells can promote tumor cell proliferation by inhibiting the activity of CD8^+^ T cells and promote metastasis. Tumor endothelial cells (TEC) and cancer-associated fibroblasts (CAF) can promote tumor proliferation and metastasis by activating survival signaling pathways and producing pro-tumorigenic cytokines. Extracellular matrix acts as a cytokine reservoir at both primary tumor site and metastasis site to support tumor growth

The concept of TME was first presented by Stephen Paget, known as the seed and soil hypothesis [67]. Paget described that the spread of tumor cells is caused by the interaction and cooperation of cancer cells (the “seed”) and the microenvironment of specific organs (the “soil”). Over the last several decades, extensive studies have refined our understanding of TME and its role in promoting tumor progression, metastasis, and its involvement in drug resistance. The TME of NPC represents a highly complex niche consisting of tumor-associated cells and non-cellular components (Fig. 2). Cellular components include tumor endothelial cells (TECs), cancer-associated fibroblasts (CAFs), and tumor-infiltrating immune cells. These components can differentially influence tumor initiation, progression, invasion, and metastasis. Furthermore, EBV infection largely contributes to the immunosuppressive TME observed in NPC. The next sections summarize the significant components of TME and the crosstalk between different cell populations.

### EBV infection in NPC

NPC is unique among head and neck cancers due to the strong causative association with EBV. EBV is known to exhibit type II latency program in NPC, where the infected cells express a specific set of viral genes including LMP1, LMP2A/B, EBNA1, EBERs and *Bam*HI-A rightward transcripts (BARTs). Specifically, the expression of LMP1 in cells exerts a broad spectrum of effects, such as increasing the levels of anti-apoptotic proteins, triggering cytokine production, and activating signaling pathways (NF-κB, PI3K, ERK-MAPK) in a ligand-independent manner [68, 69]. EBNA1 is the viral encoded DNA binding protein essential for the stable maintenance of EBV as an episome during latent infection. EBNA1 is expressed in all EBV-positive tumors and has been found to enhance cell survival [70]. It has been shown that EBV-positive NPC exhibits a higher immunosuppressive TME compared to EBV-negative NPC [71]. Recently, a comprehensive whole-genome profiling analysis of 70 NPC samples reveals multiple cell-virus interactions which are involved in NPC tumorigenesis [72]. In EBV-positive NPC, LMP1 or host somatic alterations underpin constitutive NF-κB activation. This finding, in agreement with other sequencing analysis [73] and CRISPR-based screening [74], conclude that NF-κB signaling pathway plays a central role in NPC development. Activated NF-kB results in an increased production of IL-6, IL-8, and leukemia inhibitory factor (LIF) that facilitate the recruitment of immune cells to establish a chronic and non-specific inflammation niche [75]. Interestingly, such inflammation is mainly characterized by the prevalence of macrophages and granulocytes rather than DCs, resulting in a reduced tumor antigen presentation environment and a decreased activation of T lymphocytes and NK cells allowing immune escape of EBV infected cells.

In contrast, the TME of EBV-negative NPC remains inconclusive. Single cell RNA-seq by Zhao et al. reported that EBV-negative tumors have higher degree of intra-tumoral heterogeneity and increased expression of keratin genes compared to EBV-positive tumors [76]. Moreover, EBV-negative NPC displayed higher infiltration of B cells than T cells, with higher M2 macrophages and fewer regulatory T cells (Tregs) [77]. Comprehensive analysis is still needed to delineate the pathogenesis of EBV-independent NPC.

### Cellular components

#### Tumor endothelial cells

TECs are characterized by their genetic instability, which contributes to the heterogeneity that promotes therapeutic resistance, tumor progression and metastasis. They are also known to regulate cytokine secretion and angiogenesis [78]. Tumor cells require oxygen and nutrients to survive and growth, therefore angiogenesis plays a critical role in tumor progression. TECs process increased capacities of proliferation, migration, and tube-formation in response to growth factors (e.g., VEGF and FGF2) and cytokines (e.g., CXCL1 and CXCL8), which may be attributed to the increased expression of growth factor and cytokine receptors such as TEK and VEGFR [79]. In EBV-positive NPC, LMP1 was shown to induce the expression of Cyclooxygenase-2 (COX-2) via NF-κB pathway. Murono et al. reported that LMP1 can increase the production of VEGF by acting together with COX-2, thereby contributing to angiogenesis in NPC [80]. EBV can also infect endothelial cells using monocytes as a shuttle [81]. The infected endothelial cells display upregulation of EBV lytic genes, promoting inflammation and vascular injury. Cheng et al. found that TECs co-cultured with cancer cells show constitutive activation of PI3K/AKT signaling pathway, resulted in cell survival and tube formation, creating a microenvironment that favors tumor growth [82]. Inhibition of PI3K and COX-2 signaling pathways can reverse the increased tube formation and induce apoptosis of endothelial cells. More importantly, TECs are irregular monolayers with impaired barrier functions compared to the normal endothelial cells, which could lead to vascular leakiness and provide a route for metastasis [83]. With angiogenesis, tumor cells can intravasate from primary site into blood circulation and extravasate into distant organs. Even though millions of tumor cells could be released into the circulation, only a few of them can successfully form distant metastatic nodes. The reason is that inadequate or inappropriate cell–matrix interaction when tumor cells detach from ECM in primary tumor cause them to enter cell-cycle arrest and rapid apoptosis, known as anoikis. Yadav et al. demonstrated that TEC could promote tumor metastasis by chaperoning tumor cells to distal sites [84]. The activated TECs can upregulate the expression of adhesion molecules and bind to tumor cells, thereby protecting the circulating tumor cells from anoikis and facilitating their movement to distant sites.

#### Cancer-associated fibroblasts

Among the various cell types present in the TME, Cancer-Associated Fibroblasts (CAFs) have been identified as significant mediators of cancer cell migration and invasion in numerous solid tumors. The primary origins of CAFs are the local normal fibroblasts. Kojima et al. found that tumor cells can produce TGF-β and SDF-1 to initiate and maintain the transformation of normal fibroblasts to CAFs. In addition, Kalluri et al. described that the endothelial cells may undergo endothelial-mesenchymal transition and convert to fibroblast-like cells, which accounts for a large proportion of CAFs [85]. Importantly, EBV-encoded LMP1 has been demonstrated to promote the transition of normal fibroblast to CAFs. Wu et al. reported that extracellular vesicles (EVs) packaged LMP1 can regulate CAF transition via activation of the NF-κB RelA/p65 signaling pathway [86]. Co-culture of EVs-activated CAFs enhances the proliferation, migration, and radiation resistance of NPC cell line HK1. A study by Davis et al. revealed another mechanism in which LMP1 transforms fibroblasts [87]. They showed that conditioned medium from MDCK-LMP1 cells increases cell motility and invasion in both epithelial and fibroblasts by stimulating TGF-β and ERK-MAPK signaling pathways. However, since these pathways have extensive crosstalk with other pathways and regulatory factors, the precise mechanism is unclear and requires further investigation. Functionally, CAFs are documented to enhance angiogenesis in tumors due to their large production of VEGFA within the TME [88]. Furthermore, it has been documented that CAFs maintain the capacity to stimulate angiogenesis independently of tumor cells, as they respond to PDGF-C. [89]. In addition, CAFs can promote tumor angiogenesis by recruiting endothelial progenitor cells via CXCL12/CXCR4 signaling pathway [90]. In NPC, CAFs are found to facilitate tumor migration and invasion, leading to worse prognosis. In support of this notion CAFs promote survival and confer RT resistance in NPC cells by secreting IL-8, which activates NF-kB signaling pathway and reduces DNA damage [91].

#### Tumor-infiltrating immune cells

TILs are immune cells triggered by the host's immune response to fight against the tumor, including lymphocytes, DCs, macrophages, and mast cells. However, cancer cells possess a wide array of mechanisms to circumvent the immune response through an intricate and ever-changing interactions with TILs, promoting tumor progression, metastasis, and resistance to various drugs [92]. Therefore, TILs serve the host immune defense but can be hijacked by cancer cells to provide local support for the tumor.

##### T lymphocytes

The most significant mediators of adaptive immune response are the T lymphocytes which can recognize and eliminate cancer cells. The cytotoxic CD8^+^ T cells, along with CD4^+^ T helper type 1 (Th1) cells are the final effector cells for tumor elimination [93]. On the contrary, the CD4^+^ T cell subsets Th2 and Th17 are usually associated with pro-tumorigenic activity. However, the role of Th17 in the tumor immunity remained controversial and has been associated with both favorable and unfavorable outcomes [94]. In EBV-positive tumors, the elevated presence of CD8^+^ T cells is indicative of a greater proportion of effector T cells that express cytotoxic molecules. Nevertheless, EBV possesses the capability to encode proteins and small non-coding RNAs to suppress the expression of Human Leukocyte Antigen class I (HLA I) antigens, thus disrupting the antigen presentation process. This evasion strategy enables EBV-infected cells to avoid recognition by CD8^+^ T cells [95]. Moreover, the CD8-mediated immune response can be suppressed by Foxp3/CD25 positive regulatory T cells (Tregs). Gondhowiardjo and colleagues conducted an analysis of biopsy samples from 23 patients with NPC and found a positive association between larger primary NPC tumors and higher CD8 marker expression [71]. However, despite an abundant CD8^+^ T cells in the nasopharynx it is believed that these cells are dysfunctional. Notably, a significant correlation between Foxp3 expression and tumor volume was found. Tregs, which share many molecular signaling pathways with conventional T cells and play a crucial role in preventing autoimmunity, were found to impede anti-tumor immunity. In another study, analyses of T cell subsets at various stages of NPC revealed that Tregs were notably upregulated in NPC patients who were previously untreated, in partial remission, and in the relapse groups [96]. Since the majority of NPC immune cells express PD-L1, it is believed that the expression of PD-L1 on Treg could inhibit tumor immunity and promote immune escape of cancer cells [97]. The increased level of Tregs not only interferes antitumor immune response but may also represent a major obstacle to immunotherapy treatments [98]. In fact, aggregation of Tregs in the TME may predict poor prognosis in some tumors [99]. Progress in our understanding of Treg functions provides a basis for considering Treg depletion and the regulation of Treg function as strategies to bolster the immune response. Nevertheless, neutralizing Tregs to amplify anti-tumor immune responses comes at the potential cost of triggering autoimmunity. Therefore, the major challenge is to specifically deplete tumor infiltrating Tregs without affecting effector T cells.

##### B lymphocytes

Tumor-infiltrating B cells represent a large heterogeneous group of cells with diverse functions in the TME. Activated B cells can differentiate into plasma cells and produce antibodies to label pathogens or infected cells for the host immune defense. While accumulating data has strongly indicated a critical role for B cells in antitumor immunity, the function of B cells in this process remains inconclusive. Wouters and colleagues conducted a comprehensive review of sixty-nine clinical studies encompassing 19 cancer types. Their findings suggested a prognostic impact of tumor-infiltrating B cells in various cancers, including breast cancer, colorectal cancer, esophageal cancer, melanoma, among others [76]. However, a separate genomic analysis indicated that elevated expression of B cell metagenes was associated with poorer survival outcomes in patients with clear cell renal tumors [100]. Recent studies suggest that B cells may serve as effective promoters of metastasis through IL-1β/HIF-2α/Notch1 signaling pathway which stimulate cancer cell migration and invasion [101]. Single-cell analysis in NPC revealed that high abundance of intratumoral B cells is associated with better prognosis in NPC patients. On the contrary, the abundance of IgD^−^CD27^−^ double-negative B cells was significantly correlated to worsen prognosis of NPC patients [102]. Double-negative B cells are a small subset of B-cell population, which has been shown to involve in various diseases, including malaria, chronic inflammatory disorders, non-small cell lung cancer and NPC [103]. Since EBV infection is strongly associated with NPC, the virus may also crosstalk with B cells to suppress immune activity. Along these lines, EBV LMP1 can block B cell differentiation and EBV-infected cells express miR-21 [104, 105] which can increase the expression of immunosuppressive cytokine IL-10, suppressing cytotoxic CD8^+^ T cell activities. These observations suggest that B cells likely play an important role in regulating the outcome of radio-chemotherapy and immunotherapy in NPC.

##### Natural killer cells

Natural killer (NK) cells are innate cytotoxic lymphoid cells that can actively prevent tumor growth via immunosurveillance. NK cells can be divided into two populations based on expression levels of CD56 and CD16. About 90% of NK cells are CD56^dim^CD16 + with high cytotoxic potential and 10% are CD56^bright^CD16^−^ with poor cytotoxic potential. In cancer patients, NK cells can recognize and eliminate tumor cells by releasing cytolytic molecules perforin and granzymes, as well as modulate the adaptive anti-tumor immune response by producing chemokines and cytokines such as IFN-γ, TNF-α, IL-8, IL-10, and CCL2 [106]. Previous studies showed that the NK cell killing effect predominately relies on TNF-induced apoptosis [107]. In addition, concomitant treatment with IFN-β and PD-1/PD-L1 checkpoint blockage could further increases the killing effect on NPC cells. Recently, Glasner et al. reported a mechanism by which NK cells exert their antitumor effect. Investigations revealed that human NK cell receptor NKp46 can induce IFN-γ production from intratumoral NK cells, upregulating the expression of fibronectin 1 in the tumors and preventing metastases formation [108]. Compelling evidence reveal that the TME can negatively modulate NK cell functionality by producing immunosuppressive factors (i.e., TGF-β, PGE2, IDO) and decrease the antitumor effect of NK cells [109, 110]. Particularly, the NK cell functions are impaired when the EBV-infected cells enter the latency phase [111]. During latency phase, EBV produces viral proteins to evade NK killing, such as LMP1, LMP2A/B. For example, LMP1 is shown to cause resistance to NK-mediated cell death by upregulating anti-apoptotic proteins Bcl2, surviving, and pro-survival receptor 4-1BB [112, 113]. EBV also stimulates production of cellular proteins such as anti-apoptosis Bcl2, and immunosuppressant IL-10.

##### Macrophages

Tumor-associated macrophages (TAMs) are the largest immune cell population within the tumor stroma, with dual supportive and suppressive role in cancer. Early findings showed that macrophages activated by cytokines are capable to eradicate cancer cells [114]. However, TAMs can rapidly loose the cytotoxic activity and instead begin stimulating tumor growth and metastasis [115]. To date, it is established that TAMs can polarize into different phenotype in response to cytokines and the microenvironment [116]. The maturation to M1 macrophage phenotypes is driven by IFN-γ, TNF-α and microbial products, shaping the cells with antitumor activity. On the contrary, M2 macrophages derived from exposure to IL-4 or IL-13. These macrophages, with the expression of surface marker CD162 and CD206, can stimulate angiogenesis, suppress immunity, and promote tumor growth and metastasis [117]. Wang et al. showed that NPC cell-derived FGF2 can increase pericytes proliferation and the expression of CXCL14, which leads to the recruitment and polarization of TAMs and TAM-associated metastasis [118]. More importantly, TAMs can regulate the behavior of cancer cells and the TME. Cancer cells recruit TAMs by secreting colony-stimulating factor-1, and in return, TAMs facilitate cancer cell growth by producing EGF [119]. TAMs contribute to cancer metastasis by disassembling the extracellular matrix of the TME, allowing cancer cells to disseminate from the primary tumor site. Moreover, at distant sites, Tumor-Associated Macrophages (TAMs) have the capability to establish a protective environment for metastatic cancer cells, facilitating their accumulation through the secretion of interleukin-1 (IL-1) [117]. Interestingly, macrophages in NPC have been shown to exhibit distinctive M1-M2 coupled pattern, indicating an intermediate phenotype between tumor suppressive and tumor promotive subtypes [120]. Such paradoxical activity may be associated with chronic EBV infection, which induces long-lasting inflammatory responses in the TME.

#### Extracellular matrix

TME also includes a non-cellular component known as the extracellular matrix (ECM). All the tumor cells, non-cancerous cells and immune cells are interacting within the ECM. ECM is a complex network composed of fibrous proteins, matrix proteins, and proteoglycans that provides structural support and facilitates tumor progression, intravasation, and metastasis [121]. It serves as a reservoir for various nearby cell-secreted cytokines, hormones, and pro-angiogenic growth factors, playing a significant role in angiogenesis and vascular stabilization [122]. Another key feature of ECM is the ability to help tumor cells escape immune surveillance. It is well-documented that ECM can modulate the activity of lymphocytes, such as migration, recognition/activation, and differentiation [123]. ECM deposition and increased stiffness can reduce lymphocyte replacement, limit T cell infiltration, and therefore providing a more favorable environment for tumor cell growth [124]. Because of the rapid tumor expansion and poor vascularization, the ECM is recognized as a hypoxic environment. In response to these conditions, tumor cells undergo a metabolic transition from oxidative phosphorylation to glycolysis. Recent findings suggest that the tumor-imposed glycolysis metabolism can restrict T cell functions, dampening the mTOR activity, glycolytic capacity, and IFN-γ production, causing T cell hypo-responsiveness during cancer [125].

### Noncellular components

#### Cytokines and chemokines

The secretion of proinflammatory and immunosuppressive cytokines and chemokines can have a significant impact on immune cell activity and modulate the TME for tumor cell proliferation, immune escape, and apoptosis resistance. A multiplexed immuno-based profiling of cytokine markers in NPC [126] revealed elevated levels of IL-6, IL-8, TNF-α, VEGF, CXCL-10, and MIP-3α in NPC compared to healthy individuals. Moreover, the elevated cytokine levels were correlated with EBV DNA and demonstrated worse prognoses for OS. Several studies have shown that IL-6 and its receptor are broadly expressed among NPC cell lines [127]. IL-6 can promote NPC cell migration and invasion, which may be mediated by the regulation of MMP-2 and MMP-9. Moreover, the use of anti-human IL-6R antibody significantly inhibited NPC cell growth and invasion, suggesting blockade of IL6/IL6R is a potential therapeutic target to treat NPC metastasis. Several studies have found that IL-8 promoter T-251 T/A genetic variation is significantly associated to NPC risk [128–130]. Compared to TT genotype, AA and AT genotypes were highly associated with susceptibility and aggressiveness of NPC, suggesting an important role of IL-8 in NPC progression. Along these lines, IL-8 can stimulate angiogenesis increasing blood supply to the tumor, promoting its growth and survival. The inflammatory cytokine TNF-α is recognized for its dual role in cancer, which depends on factors such as its concentration, duration of exposure, and the presence of other chemokines or cytokines within the TME. Short-term, localized administration of TNF-α has been demonstrated to exhibit anti-tumor effects, whereas prolonged expression of TNF-α can lead to a pro-tumorigenic state [131]. A multivariate analysis by Yu et al. revealed that elevated expression of TNF-α in primary NPC tissues was associated with increased risk of distant metastasis, particularly bone metastasis [132]. NPC cell lines CNE-2 and HK-1, as well as NPC biopsies, express IL-18 and CXCL-10 [133]. IL-18 can induce IFN-γ production from T cells and NK cells, which can lead to the activation of macrophages and other immune cells to secrete chemokines to initiate immune cell recruitment. Additional studies suggest that the elevated IL-18 level in TME can induce PD-1 expression on NK cells, a marker of functional exhaustion [134]. Therefore, despite the higher percentages for infiltrated NK cells, the functional exhaustion mitigates their cytotoxic effect and leads to poor prognosis.

#### Metabolites

Generally, cancer cells display competitive advantage over other cells for nutrients. It has been shown that EBV-encoded LMP1 can modulate NPC energy metabolism via the FGFR1 signaling pathway, characterized by increased glucose and glutamine consumption, LDHA activity, lactate production, and secretion of HIF-1α [69, 135]. LMP1 acts by upregulating the expression of FGFR1 in NPC cells through multiple downstream targets and signaling pathways. Consequently, the constitutive FGFR1 activation facilitates LMP1-mediated NPC cell transformation, migration, and invasion. These studies suggest that LMP1 promotes aerobic glycolysis through the regulation of metabolic enzymes and related genes. Since TILs also rely on glucose, it is believed that insufficient glucose uptake could contribute to the formation of immunosuppressive TME [136, 137]. Reinfeld and colleagues found that among myeloid cells, T cells, and tumor cells, myeloid cells exhibited the most significant capacity for capturing intratumoral glucose, followed by T cells, and tumor cells [113]. Notably, tumor cells displayed the highest glutamine uptake. Tumor cells take up glutamine and convert it to glutamate through mitochondrial oxidative phosphorylation to produce energy and support their growth and metastasis. Competition for glutamine uptake also exists between tumor cells and immune cells in the TME. Studies have demonstrated that competitive consumption of glutamine by tumor cells could lead to suppressed antitumor immune response in many cancers [138, 139]. Inhibiting glutamine uptake in tumor cells could potentially enhance glucose uptake, indicating that glutamine may serve as the limiting factor in the TME [140].

Lactate is one of the tumor cell metabolites that can serve as a prognostic parameter for metastasis and overall survival of patients. Clinical study showed that high lactate level in the TME inhibits lactate export in T cells, which hinders their metabolism and function [140]. In addition, lactate could interfere with the function of DCs and TAMs, as well as inhibit monocyte migration and cytokine (TNF, IL-6) release [141]. Colegio et al. showed that tumor-derived lactic acid can act through HIF-1α to induce the expression of VEGF and the M2-like polarization of TAMs [142]. In addition, lactate promotes tumor cell proliferation by upregulating the expression of arginase 1 in TAMs. Arginase can promote tumor cell proliferation by depleting arginine from the microenvironment, inducing arginine autotrophy in certain tumor cells, suppressing the immune response, and contributing to an immunosuppressive tumor microenvironment.

Another important metabolite of NPC cells is indoleamine 2,3-dioxygenase (IDO), a cancer-associated enzyme that catabolizes intracellular tryptophan to produce kynurenine [143]. The process of tryptophan degradation and kynurenine production together contribute to immunosuppressive effect, leading to immune escape of tumors. It is well-established that T cells are highly sensitive to tryptophan depletion, which causes a mid-G1 arrest [144]. Kynurenine in the TME can act on the ligand-activated transcription factor aryl hydrocarbon receptor to drive the differentiation of Tregs, tolerogenic myeloid cells and PD-1 upregulation in CD8^+^ T cells [145]. Moreover, selective inhibition of the receptor could impede the progression of IDO-overexpression tumors, making it a new target for immunotherapy.

#### Exosomes

Exosomes, a subset of EVs that originate from either the endosome or the cell's plasma membrane, play a pivotal role in facilitating cell-to-cell communication. They are considered one of the foremost mechanisms for establishing the diverse characteristics of the TME [146]. To date, accumulated evidence has suggested that exosomes play a significant role in NPC progression, metastasis, and resistance to therapies. In fact, NPC-derived exosomes and CNE2-derived exosomes are enriched in PFKFB3, which facilitates endothelial cell proliferation, migration, and angiogenesis through the activation of the ERK and the AKT pathways [147]. In addition, highly metastatic NPC cells could transfer EGFR-rich EV to poorly metastatic NPC cells to enhance their tumor metastasis potential [148]. Mechanistically, the EGFR-rich EV could induce EGFR upregulation and ROS downregulation via the PI3K/AKT signaling pathway. NPC cells infected with EBV can stimulate tumor progression by using the exosome system for the transfer of signaling molecules, viral proteins, and microRNAs. EBV LMP1 oncoprotein, was reported to be secreted from EBV-positive NPC cells via exosomes to modulate the TME through intracellular trafficking [149]. Studies showed that LMP1 can promote EV formation by upregulating the levels of syndecan-2 and synaptotagmin-like-4 through activation of the NF-κB signaling pathway [150]. This is further supported by investigations revealing that syndecan-2 can interact with syntenin to promote the formation of EVs, while synaptotagmin-like-4 can regulate the release of EVs. NF-κB activation is required to initiate the production of LMP1-packaged exosome, which contribute to the epithelial-mesenchymal transition (EMT) potential of EBV-negative recipient NPC cells [151]. Furthermore, inhibition of NF-κB significantly repressed exosome LMP1 secretion and limited NPC lung metastasis in nude mice. Other EBV-related exosomes include LMP2A [152], BART1 [153], HMGA2 [154]. These exosomes are found to promote tumor metastasis and modulate immunosuppressive TME.

Recently, additional evidence confirmed that TME-derived exosomes are involved in NPC development. Shi et al. found that mesenchymal stem cells (MSCs) secrete FGF19-rich exosomes [155]. Co-incubation study suggested that NPC cells can pick up the exosomes, leading to enhanced cell proliferation, migration, and tumorigenesis. Mechanistically, FGF19-rich exosomes can activate the FGF19-FGFR4-dependent ERK signaling cascade and modulate EMT process. Another study reported that MSCs could be used as a therapeutic tool in NPC. MSCs transfected with miR-34c, a tumor suppressor miRNA, could produce exosomes that attenuate NPC proliferation, migration, invasion and EMT process [156]. Notably, miR-34c-overexpressing exosomes significantly sensitized NPC cells to RT.

## Drug resistance mechanisms in NPC

### Chemotherapy

**Fig. 3:**
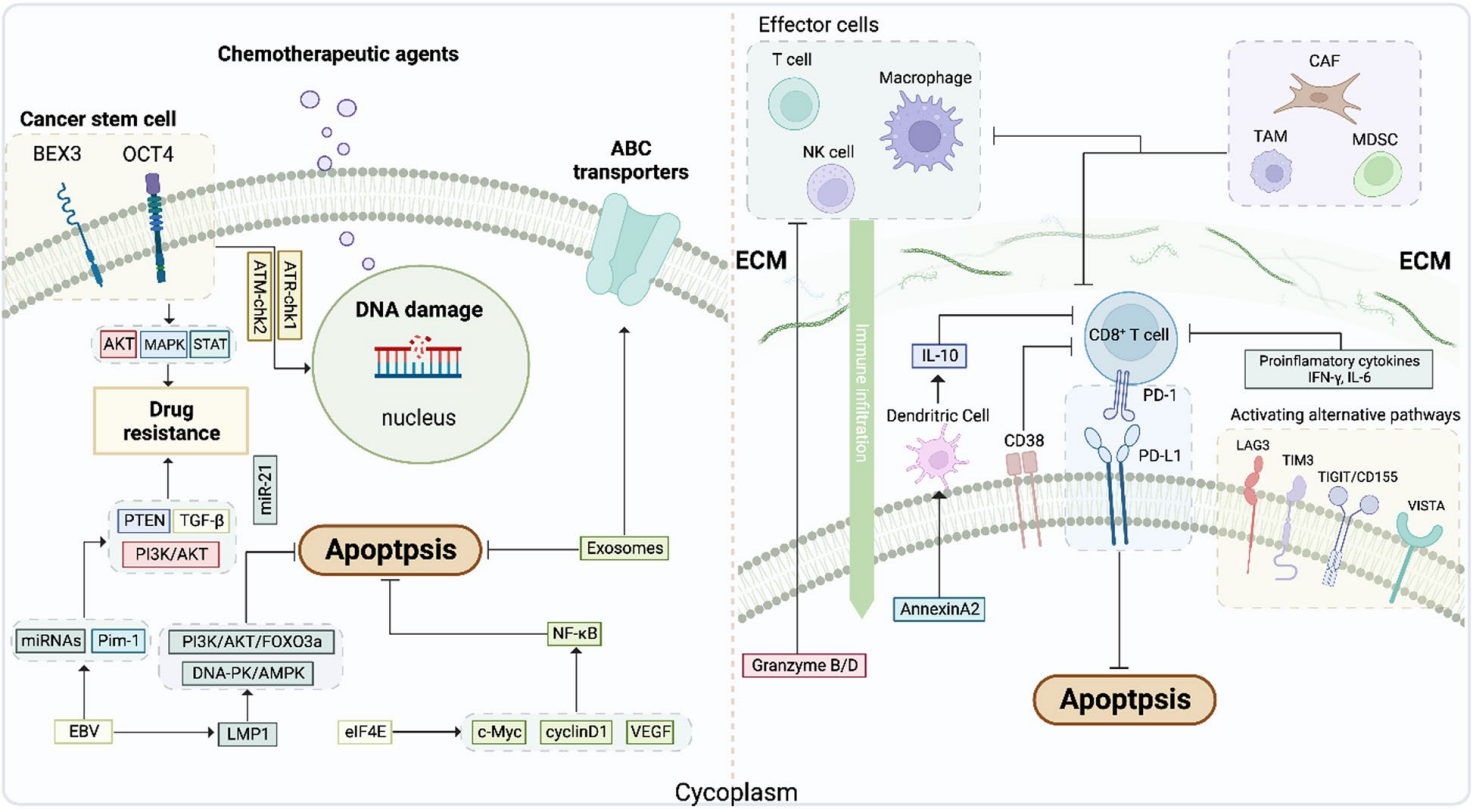
Drug resistance mechanisms of chemotherapy and immunotherapy. Nasopharyngeal carcinoma cells could develop various drug resistance mechanism against anticancer therapy. **A** Chemotherapeutic agents exert the anticancer effect through DNA damage and induce cell apoptosis. Upregulation of proteins and miRNAs can inhibit apoptosis and directly confer cell drug resistance phenotype. **B** Tumor cells can develop drug resistance towards immunotherapy through the interaction with tumor microenvironment. Immune effector cells are able to identify and kill tumor cells through immune infiltration into the tumor site. Granzyme B and D can inhibit the immune infiltration process. Annexin A2 can stimulate Dendritic cells to produce IL-10, which has inhibitory effect on cytotoxic CD8^+^ T cells. Pro-inflammatory cytokines IL-6 and IFN-γ can decrease CD4/CD8 T cell ratio. Cancer-associated fibroblasts (CAF), tumor-associated macrophages (TAM), and myeloid derived suppressor cells (MDSC) can inhibit the activity of immune effector cells. Tumor cells can activate alternative pathways to circumvent PD-1/PD-L1 blockade

Despite the controversy over the efficacy of adjuvant chemotherapy in patients with NPC, the use of platinum-based multidrug chemotherapy remains the standard treatment for recurrent NPC. In addition, IMRT is another current standard of care for NPC, and has shown promising results for PFS and OS in early-stage patients. However, the development of chemoradiation drug resistance is a major obstacle for recurrent NPC patients [157]. Besides tumor cell-related drug mechanisms, changes in the TME are important factors contributing to chemoradiation resistance in NPC (Fig. 3).

#### EBV-related mechanisms

NPC is a cancer whose pathogenesis is highly correlated with EBV, therefore EBV protein and miRNA are thought to be one of the mechanisms of chemoresistance in NPC patients [158]. LMP1, a transmembrane protein of the EBV virus, is found to be expressed in 70% of NPC. It can induce chemoresistance by sequestering Pim-1 in the cytoplasm or activating the PI3K/AKT signaling pathway [159, 160]. Yang et al. reported that LMP1 can upregulate the expression of miRNA-21 via the PI3K/AKT/FOXO3a signaling pathway. In turn, high level of miR-21 could lead to cisplatin resistance through the inhibition of pro-apoptotic factors PDCD4 and FasL [161]. Meanwhile, studies also show that LMP1 can inhibit the DNA damage response through DNA-PK/AMPK signaling, and LMP1 positively regulates the expression of the cancer stem cell (CSC) marker CD44 and several stemness-associated genes (Nanog, Oct4, Bmi-1, and SOX2), which induces the development of CSCs and contributes to RT resistance in NPC [162, 163].

Among the EBV virally-encoded miRNAs, some are associated with chemotherapeutic drug resistance, such as miR-BART22, miR-BART7-3p, and miRBART5; while others are associated with RT resistance, miR-BART4, miR-BART7, and miR-BART8-3p [164–166]. Studies have revealed that miR-BART22 promotes MYH9 expression through the PI3K/AKT/c-Jun pathway, leading to EMT and cisplatin resistance in vitro and in vivo [167]. Others have reported that miR-BART7-3p inhibits PTEN, activates the PI3K/AKT/GSK-3β signaling pathway, and promotes the expression and nuclear aggregation of β-catenin, which in turns facilitates EMT [168]. Similarly, another study demonstrated that miRBART7-3p targets SMAD7 and triggers the TGF-β pathway, inducing cancer stem-like cells and chemoresistance to fluorouracil, cisplatin, and paclitaxel [169]. By downregulating p53 and simultaneously up-regulating apoptosis regulator, miR-BART5 expression reduces the sensitivity of NPC cells to pro-apoptotic drugs such as doxorubicin and etoposide [170].

#### Abnormal DNA damage repair

Cisplatin is one of the standard treatments for patients with advanced NPC. Cisplatin has the capability to engage with the DNA within tumor cells, creating both intra-strand and inter-strand links. This interference disrupts DNA replication and transcription processes, ultimately leading to the destruction of tumor cells [171]. Consequently, abnormal DNA damage repair function is one of the main mechanisms of cisplatin resistance. FOX family is a group of evolutionarily conserved proteins that can bind to DNA to regulate transcription [172]. The transcription factor FoxM1 is a critical proliferation-associated transcription factor that is widely spatiotemporally expressed during the cell cycle [173], and FOXM1, FOXC2 and FOXQ1 have been reported to be overexpressed and involved in the occurrence of EMT and chemoresistance in NPC [174, 175]. Studies have shown that downregulation of FoxM1 inhibited MRN-ATM-mediated DNA repair and subsequently increased the sensitivity of NPC cells to cisplatin. Another study revealed that MRN-ATM-mediated DNA repair was inhibited by the downregulation of FOXM1, which subsequently increased the sensitivity of NPC cells to cisplatin [176]. In addition, a specific non-coding RNA, the circular RNA CircCRIM1, was found to be overexpressed in NPC cells to suppress the inhibitory effect of miR-422a on FOXQ1, thereby inducing chemoresistance towards docetaxel [177]. It has been reported that circRNAs are abundant and stably expressed in exosomes and play critical roles in mediating chemotherapy resistance in various cancers [178, 179]. Therefore, further study is warranty to study the role of other circRNAs in NPC drug resistance.

#### ATP-binding cassette transporter

The ATP-binding cassette (ABC) transporter is a superfamily of transmembrane proteins, which are widely expressed in cell membranes [180]. Its involvement in anti-cancer drug efflux is a common cause of chemoresistance [181]. Currently, ABCB1/P-gp and ABCC1/MRP1, ABCC5, and ABCG2/BCRP have been found to be overexpressed in NPC drug-resistant cells [174, 182]. ABC transporters have the capacity to expel a broad spectrum of anticancer drugs from within cells, giving rise to a multidrug resistance phenotype in cancer cells [183]. For instance, ABCB1 and ABCG2 are key resistance factor to chemotherapeutic drugs paclitaxel and docetaxel, as well as EGFR inhibitors gefitinib and erlotinib. Notably, there is a strong association between cancer stem cells and the emergence of chemoresistance [184, 185], and in fact both ABCG2 and ABCB1 are expressed in cancer stem cells. The isolated cancer-stem like side population cells from NPC demonstrated increased expression of ABCG2 and the anti-apoptotic factor Bmi-1. These factors play a role in enhancing multidrug resistance and elevating the survival rate of tumor cells [186]. Therefore, the evidence provides a reference for the use of chemotherapy in NPC.

#### Inhibition of NPC cell apoptosis

Another common chemo-resistance mechanism is the inhibition of apoptosis in NPC cells [187, 188]. Eukaryotic translation initiation factor 4E (eIF4E) has long been recognized as a quantitative restriction initiator of mRNA translation. Genome-wide analysis by Truitt et al. showed that elF4E dosage plays a critical role in the translational program induced by oncogenic transformation [189]. Compared to parental cells, high expression of eIF4E was found in cisplatin-resistant NPC cells, which promoted the translation of c-Myc, cell cycle protein D1, and VEGF to circumvent drug-induced apoptosis. In addition, in vitro and in vivo studies have confirmed that TBL1XR1, an essential transcriptional cofactor, can promote anti-apoptosis activity by activating the NF-κB pathway. Hence, its high expression can trigger NPC cells to become resistant to cisplatin-induced apoptosis [190].

#### CSCs and EMT

CSCs are cells within a tumor that can self-renew and form heterogeneous cell populations that lead to heterogeneous branching of cancer cells in the tumor. It can be characterized using different cancer stem cell markers [191]. BEX3, a receptor-associated protein for the CSC marker CD271, is overexpressed in cisplatin-resistant NPC cells and is associated with drug resistance through the activation of the MAPK pathway [192]. In the cisplatin-resistant NPC xenograft, treatment with nontoxic level of cisplatin led to significantly increase in BEX3 level. In addition, BEX3 can induce the expression of OCT-4, another cancer stem cell marker, which contributes to chemoresistance through activation of the AKT pathway and the STAT3 pathway [193]. In addition, CSCs can also induce drug resistance by scavenging ROS from oxidative stress, activating anti-apoptotic pathways, and protecting the microenvironmental niche [194]. The phenotype of CSCs may be closely related to EMT, which renders epithelial cells mesenchymal properties characterized by the loss of epithelial markers (e.g., E-cadherin, α-cadherin) and the gain of mesenchymal markers (e.g., Vimentin, fibronectin, N-cadherin). EMT process not only enhances tumor cell migration and invasion, but also induces drug resistance to chemotherapy.

Recently, the TGF-β pathway has been implicated in chemoresistance through EMT or maintenance of tumor-induced cell heterogeneity [195]. In vitro study shown that shRNA-mediated downregulation of TGF-β induces phosphorylation of PTEN and AKT, thereby increasing cisplatin resistance. In contrast, overexpression of TGF-β sensitized NPC cells to cisplatin. In fact, out of the four identified miRNAs associated with an increased risk of advanced nasopharyngeal cancer, MiR-449b has the capacity to modify the TGF-β pathway, leading to the development of cisplatin resistance in NPC [196].

#### Exosomes

Exosomes are EVs that carry information including proteins, mRNAs, miRNAs, and function as intercellular communication [197]. Co-incubation of doxorubicin-resistant human microvascular endothelial cells-derived exosomes with NPC cells can induce the proliferation, migration, EMT, and the development of chemotherapy resistance in NPC cells [198]. Moreover, the exosomes induced the expression level of ABC transporters ABCB1 and ABCC1 that are capable of causing multidrug resistance. In addition, other studies have demonstrated that LMP1-positive exosomes extracted from EBV-infected NPC cells can promote chemoradiation resistance [86]. The specific regulatory pathways related to drug resistance remain to be elucidated and warrant future studies.

### Immunotherapy

#### Immune checkpoint inhibitors (ICI)

Despite the encouraging efficacy of anti-PD-1/PD-L1 therapy, many patients failed to response or developed acquired resistance towards ICIs [199, 200]. Potential drug resistance mechanisms toward ICIs can result from impaired T-cell proliferation and functions. In fact, genetic alteration in granzyme, gasdermin, and IFN were enriched in refractory NPC tumors [201]. Specifically, the granzymes GZMD and GZMB are known to induce apoptosis of cytotoxic T cells and NK cells. Cancer cells resistance to ICIs treatment may occur through the dysfunctional pyroptosis pathway and weakened cytotoxic lymphocyte function. Using an NPC-PDX mouse model to test the combination of anti-PD-1 antibody nivolumab and anti-CTLA-4 antibody ipilimumab [202]. Interestingly, human proinflammatory cytokines including IFN-γ and IL-6 were significantly upregulated in plasma, accompanied by a decrease in CD4/CD8 ratio. Moreover, the isolated TILs were responsive to stimulation but remain insufficient to induce antitumor effect in vivo. Drug resistance was observed in this NPC-PDX model, which could be mediated by other inhibitory checkpoints such as LAG3 and TIM3 as well as immunosuppressive molecules in the TME [202].

Others have reported activation of alternative immune checkpoints as drug resistance mechanisms to PD-1/PD-L1 blockade. The alternative pathways include LAG3, TIM3, TIGIT/CD155, and VISTA [203]. In addition, adenosine receptor signaling can suppress various immune cells to create an immunosuppressive niche. Chen et al. reported that tumor cells could develop drug resistance against PD-1/PD-L1 blocking antibodies by upregulating CD38, which is induced by all-trans retinoic acid and IFN-β in the TME [204]. CD38 suppresses CD8^+^ T-cell function via adenosine receptor signaling and therefore represents a major mechanism of acquired immunotherapy resistance. Long non-coding RNAs (lncRNAs) with immune-related functions in the TME have been observed to influence both immune cell infiltration and the response of cancer cells to anti-PD-1 immunotherapy [183]. In a comprehensive analysis of the whole genome expression, Tang and colleagues discovered a notable correlation between the lncRNA AFAP1-AS1 and the expression of PD-1 in patients with NPC [205]. Co-expression of AFAP1-AS1 and PD-1 in TILs predicted the poorest prognosis and distant metastasis at relapse.

The resistance to ICIs could also be attributed to the establishment of immunosuppressive tumor microenvironment resulting from immune modulatory effects of CAFs, TAMs, and myeloid-derived suppressor cells (MDSCs) [206]. Angiogenesis factor can directly promote immune suppression by suppressing the function of APCs and immune effector cells, or by enhancing the effect of Tregs, MDSCs, and TAMs [207]. Those immunosuppressive cells can reciprocatively drive angiogenesis to create a viscous cycle of impaired immune activation. Interestingly, expression of annexin A2 on NPC cells can lead to immunosuppressive responses by interacting with DCs [208]. Annexin A2 acts as a ligand for DC-SIGN DCs and therefore activates DCs to release extremely high levels of IL-10. The release of IL-10 into TME causes immunosuppressive responses including CD8^+^ T cell dysfunction, Treg expansion, and inhibition of proinflammatory IL-12 [59].

#### Adopted cell therapy

The understanding of ACT resistance mechanism remains limited, and more research is needed given the important impact TME has on antitumor immunity. Along these lines, increased frequency of MDSCs can affect the outcome of EBV-specific T cell therapy in NPC [209]. Indeed, MDSCs can expand in response to cytokine stimulation under pathological conditions and cause antigen specific or non-specific suppression of T-cell response [210]. In addition, the limited ACT effectiveness can be caused by a failure of tumor specific CTLs to expand or to survive in vivo. Tumor cells expressing Fas can utilize the Fas/FasL pathway to induce T cell apoptosis and escape immune defenses [211]. Dotti et al. reported that EBV-CTLs are highly sensitive to Fas/FasL-mediated apoptosis and that knockdown of Fas significantly improve the efficacy of EBV-CTLs in NPC and Hodgkin lymphoma [212].

## Strategies to overcome drug resistance in NPC

The mechanisms of drug resistance in NPC are determined by several factors, and the strategies to overcome drug resistance have been extensively investigated. In this section, we discuss the different approaches that have been used to reverse drug resistance by targeting the cancer cells or TME.

### Chemotherapy

NPC cells may develop chemotherapy resistance by deregulation of gene expression and alteration of signaling pathways [213]. Given the heterogeneity nature of the tumor, combination treatments are highly preferred to circumvent the development of new resistance mutations or drug resistant subclones. CRISPR/Cas9 screen identified that HMGB1 could promote DNA repair efficiency, resulting in resistance to cisplatin [214]. Other researchers also reported that HMGB1 is upregulated by EBV and promote NPC cell proliferation [215]. Inhibition of HMGB1 by glycyrrhizin, a saponin found in licorice root, could effectively impair DNA binding of HMGB1 and enhance therapeutic efficacy by 2–3 folds in vitro [214].

Recently, the utilization of miRNA-based therapy has emerged as a promising strategy to overcome chemotherapy resistance, enhancing treatment responses and ultimately improving cure rates [216]. The function of miRNAs is largely influenced by the expression of their main targets. miRNAs can serve dual role, as oncogenes or as tumor suppressors, depending on the cell types. In the context of NPC, miRNAs expression is deregulated during chemotherapy, and some miRNAs were shown to enhance the sensitivity of NPC cells to treatment. Zhang and colleagues uncovered that decreased expression of miR-29c served as a predictive marker for therapeutic resistance in a cohort of 159 NPC patients. Restoration of miR-29c significantly enhanced the sensitivity of NPC cells to cisplatin, by downregulating the expression of antiapoptotic factors Mcl-1 and Bcl-2 [217]. Interestingly, a tumor suppressor miR-3188 could induce its own expression by interacting with FOXO1 to form a mTOR/PI3K/AKT/c-JUN negative feedback loop, thereby increasing the sensitivity of NPC cells to 5-FU.

A major cause of chemoresistance is the expression of MDR-associated ABC transporters, including ABCB1, ABCG2, and ABCC1 [218]. The combination of ABC transporter inhibitors with chemotherapeutic drugs have been shown to effectively overcome the MDR activity in preclinical models [219]. However, even with the development of third generation ABCB1 inhibitor, the clinical attempts to directly block ABCB1 activity were still unsuccessful. Recently, many small molecule inhibitors have been reported to antagonize the function of ABC transporters and demonstrated antitumor efficacy. With an established PK/PD and safety profile, these inhibitors could be promising candidates to overcome MDR mediated by ABC transporters. For example, FLT3 inhibitor midostaurin can increase the accumulation of daunorubicin in peripheral blood mononuclear cells of primary CD34^+^ AML patients and those not achieving CR [220]. Moreover, the ABCB1 inhibition was observed independent of FLT3 mutation, suggesting therapeutic value of midostaurin for drug-resistant patients. In addition, MET inhibitor tepotinib can effectively inhibit the activity of ABCB1 and ABCG2 simultaneously through its interaction with the drug-binding pocket of the transporters [183, 221]. Evidence suggests that a cooperative and potentially compensatory role for ABCB1 and ABCG2 in some cancers, using drugs that can target multiple transporters may provide additional benefits compared to single inhibition [222].

Development of the EMT phenotype is one of the major mechanisms that allow tumor cells to become resistant to platinum-based drug [223]. Since cisplatin is the first-line option for NPC chemotherapy, exploiting treatment to prevent or reverse EMT is of great importance. As aforementioned, signaling pathways including Wnt, NF-κB, Notch, and TGF-β are involved in platinum-induced EMT process. Therefore, blocking these signaling pathways could affect the development of EMT in NPC cells. A recent study by Zhou et al. demonstrated that SATB1 could regulate chemoradiation resistance in NPC [224]. Knockdown of SATB1 decreases the chemotherapy resistance of NPC cells to cisplatin, suggesting a potential therapeutic target for aggressive and resistant NPC tumor. Targeting EIF4R/Snail axis can significantly increase the mRNA level of E-cadherin and sensitize NPC cells to cisplatin in invasion [225] while the Hippo pathway contributes to cisplatin-induced EMT and confers drug resistance phenotype to NPC cells [226]. Depletion of TAZ, a key mediator in the Hippo pathway, has been shown to reverse EMT phenotypes to MET characteristics and sensitize drug resistant NPC cells to cisplatin treatment. Another study showed that quercetin can inhibit YAP to recover Hippo pathway, thereby inhibiting tumor progression and increase the chemosensitivity of drug resistant NPC cells to cisplatin [227]. Studies on other cancer types suggest that using inhibitors of PI3K, AKT, and HDAC can inhibit the formation of EMT and therefore overcome cisplatin resistance [228–230]. This evidence provides the rationale for further testing using combination of targeted inhibitor with cisplatin to overcome drug resistance in NPC.

### Targeted therapy

Several inhibitors targeting VEGFR have been used for anti-angiogenesis therapy for NPC patients. While the combination of VEGFRi with standard chemotherapy have shown promising results in both preclinical models and clinical trials, the development of drug resistance is a major challenge that restricts survival benefits [231, 232]. One strategy to overcome drug resistance is adding photodynamic therapy into anti-angiogenesis therapy. Consistent with this notion, combination of photosensitizer hypericin and angiogenesis inhibitor celebrex enhanced treatment efficacy [233]. Hypericin, on one hand, causes destruction of tumor cells by producing reactive oxygen species, while Celebrex, on the other hand, blocks hypoxia-induced VEGF upregulation. Given that anti-VEGF therapy could lead to activation of alternative angiogenic signaling pathways such as FGF, PDGF, and IL-1, these pathways may serve as potential targets for reversing drug resistance. In agreement, genetic knockdown of FGF-2 in NPC tumor cells could enhance the sensitivity of tumor cells to anti-angiogenic drugs [234]. Recent studies reported that miR-16 can suppress NPC carcinogenesis and progression by targeting FGF2 to inactivate MAPK and PI3K/AKT pathways [235]. Lenvatinib, a dual VEGFR2/FGFR1 inhibitor, demonstrated more profound efficacy compared to bevacizumab-like agent. Moreover, inhibition of IL-1, CXCR1/2, and TGF-β signaling pathways mitigated the acquired resistance to anti-VEGF treatment in murine model [236]. Further investigation is required to confirm the viability of these strategies in clinical settings.

### Immunotherapy

NPC tumors could develop resistance to PD-1/PD-L1 inhibitors through multiple mechanisms, including T cell dysfunction, low tumor immunogenicity, and ncRNA modulation [237, 238]. In order to overcome PD-1/PD-L1 resistance, one needs to identify new targets as alternatives for immunotherapy. Chen et al. reported a significant increase of Galectin-9 in NPC tumor cells in recurrent NPC patients. In addition, they identified a positive correlation between high Galectin-9 expression and high TIM3/ Foxp3, and low CD8 expression on lymphocytes. Low CD8 expression is considered an independent risk factor for PFS and OS [239]. Correspondingly, Yang and colleagues elucidated the mechanism behind Galectin-9-mediated adaptive immune resistance within the TME. They found that IFN-β and IFN-γ elevate Galectin-9 expression, subsequently impeding the antitumor response by promoting apoptosis in T cells. Inhibiting Galectin-9 selectively restored the immune function of infiltrating T lymphocytes by disrupting the interaction between PD-1 and TIM3 [240]. Given that Galectin-9 is specifically expressed by NPC cells, Galectin-9/TIM3/Foxp3 interaction may serve as a potential target for overcoming PD-1/PD-L1 drug resistance. Cytokines and chemokines, such as TGF-β and LMP1, within the TME have been reported as potential targets to enhance therapeutic efficacy and overcome immunotherapy resistance [241, 242]. Ma et al. revealed that TGF-β1 could activate c-Jun/STT3A signaling pathway to promote N-glycosylation of PD-L1, thus allowing immune evasion and hampering the efficacy of PD-1/PD-L1 inhibition in NPC cells. Under this context, a clinical trial is ongoing to investigate the efficacy of TGF-β resistant CTL (NCT02065362). Dominant negative receptor is introduced into NPC-specific T cells to make them resistant to TGF-β and then determine the safety as well as the immune function of engineered CTLs. Thus, targeting TGF-β1 pathway represents a promising approach to enhance immune checkpoint blockade and overcome drug resistance [243].

Identifying biomarkers holds significance in patient selection and forecasting the response to PD-1/PD-L1 therapy. Findings from the study by Herbst and colleagues indicate that both the quantity of T cells and the presence of PD-1/PD-L1 positive T cell subsets can serve as predictive factors for therapeutic effectiveness [244]. Hence, the effect of PD-1 inhibition is significant when the tumor immunosuppressive TME is mediated by PD-1 signaling. In addition, the PD-L1 expression could change in response to immune state and therapeutic treatment [245]. It is necessary to monitor these parameters during the course of treatment to circumvent drug resistance and disease recurrent.

Recently, PD-1/PD-L1 inhibitors in combination with other anticancer drugs have shown promising efficacy and manageable safety profile in patients with various cancers [246–248]. In a preclinical study, silibinin was found to downregulate PD-L1 expression by modulating HIF-1α/LDH-A mediated cell metabolism in NPC cells offering a rationale for using silibinin to overcome PD-L1 mediated NPC drug resistance [249]. A clinical trial was conducted to evaluate the combination treatment of an anti-CTLA-4 mAB IBI-310 and an investigational PD-1 inhibitor sintilimab in patients with anti-PD1/PD-L1 resistance R/M NPC (NCT04945421). The trial was completed in early 2023 and the results will be available soon. The combination of VEGFR inhibitor axitinib with avelumab is still under clinical investigation in R/M NPC to determine the efficacy and safety (NCT04562441).

Besides PD-1/PD-L1 inhibitors, ACT represents another promising cell-based immunotherapy. Recent studies show that increase in the population of MDSCs and secretion of immunosuppressive cytokines could contribute to ACT resistance. A phase II clinical trial suggested that combining chemotherapy (gemcitabine and carboplatin) with EBV-specific CTL achieved significant improvement in overall response rate and median OS for advanced stage NPC [209, 250]. With long-term follow up study, the combination demonstrated promising clinical activity in a relatively large cohort. The proposed mechanism is that chemotherapy limited the expansion of MDSCs, allowing the CTLs to traffic to site of action and carry out the cytotoxic function. Of note, another clinical trial employed EBV-specific CTL without chemotherapy resulted in a low response rate for R/M NPC [251]. Therefore, evaluation of different chemotherapeutic agents in combination with ACT may represent a new approach to increase treatment response and overcome drug resistance.

## Conclusions and future perspectives

Despite the tremendous effort in drug development, we are still facing huge difficulty in combating NPC. Due to the anatomic site and lack of specific symptoms, NPC patients usually progress to an advanced stage at the time of diagnosis. Recent breakthroughs in single-cell sequencing and multi-omics technologies have revealed the NPC microenvironment as a tumor-promoting and immuno-suppressive niche. Most of the NPC cases are associated with oncogenic EBV infection. It is well-established that EBV is involved in the whole process of NPC initiation, development, metastasis, and invasion. It also shapes the TME towards an immunosuppressive environment, facilitating tumor growth and causing drug resistance. The TME consists of cellular and acellular components that contribute together to facilitate NPC tumorigenesis and drug resistance under therapeutic intervention. The identification of cytokines, immunosuppressive cell subpopulations and signaling pathways has allowed researchers to develop new therapeutic strategies to address the problem of TME. However, most of the targets are only evaluated in preclinical studies or early-phase clinical trials. The preclinical models are not representative and cannot mimic the complex interactions within the TME and thus more advanced in vitro 3D culture systems are needed to test the therapeutic agent. In clinical settings, due to the inter-patient heterogeneity, the treatment outcomes may be highly variable among patients. There is no doubt that personalized strategies should be designed to achieve the maximum therapeutic effect with a favorable toxicity profile, which yields better outcomes for NPC patients. Moreover, drug resistance poses a substantial hurdle in anticancer treatment. A growing body of evidence suggests that TME plays a pivotal role in influencing drug responses and resistance to therapy. Hence, there is an unmet medical need to develop new approaches to circumvent or overcome drug resistance caused by TME. Combination therapies need to be optimized to tackle the progressive acquisition of drug resistance. Particularly, based on the patient’s profile, targeting the TME with novel immunotherapy to hinder tumorigenesis and restore immune surveillance could be a promising approach. In-depth clinical studies should be considered to evaluate these options and identify predictive biomarkers for personalized treatment.

## Data Availability

Not applicable.

## Abbreviations

3D-CRT: Three-dimensional conformal RT
5-FU: 5-Fluorouracil
ABC transporter: ATP-binding cassette (ABC) transporter
ACT: Adoptive cell therapy
BARTs: BamHI-A rightward transcripts
CAFs: Cancer-associated fibroblasts
COX-2: Cyclooxygenase-2
CSCs: Cancer stem cells (CSC)
CTLA-4: Cytotoxic T lymphocyte-associated protein 4
CTLs: Cytotoxic T lymphocytes
DCs: Dendritic cells
EBNAs: EBV nuclear antigens
EBV: Epstein-Barr virus
ECM: Extracellular matrix
EGFR: Epidermal growth factor receptor
eIF4E: Eukaryotic translation initiation factor 4E
ERK: Extracellular-signal-regulated kinase
EMT: Epithelial-mesenchymal transition
EVs: Extracellular vesicles
ICIs: Immune checkpoint inhibitors
IDO: 2,3-Dioxygenase
IMRT: Intensity-modulated RT
IR: Ionizing radiation
JNK: C-Jun N-terminal kinase
LMPs: Latent membrane proteins
lncRNAs: Long non-coding RNAs
MAPK: Mitogen-activated protein kinase
MDSCs: Myeloid-derived suppressor cells
MSCs: Mesenchymal stem cells
NK cells: Natural killer
NPC: Nasopharyngeal carcinoma
OS: Overall survival rate
PD-1/PD-L1: Programmed death-1/programmed death ligand-1
PFS: Progression-free survival
R/M NPC: Recurrent or metastatic nasopharyngeal carcinoma
ROS: Reactive oxygen species
RT: Radiotherapy
TAMs: Tumor-associated macrophages
TECs: Tumor endothelial cells
TILs: Tumor-Infiltrating lymphocytes
TME: Tumor microenvironment
Tregs: Regulatory T cells
VEGFR: Vascular endothelial growth factor

## References

[1] Petersson F (2015). Nasopharyngeal carcinoma: a review. Semin Diagn Pathol.

[2] Bei JX, Zuo XY, Liu WS, Guo YM, Zeng YX (2016). Genetic susceptibility to the endemic form of NPC. Chin Clin Oncol.

[3] Chen YP, Chan ATC, Le QT, Blanchard P, Sun Y, Ma J (2019). Nasopharyngeal carcinoma. Lancet.

[4] Jicman Stan D, Niculet E, Lungu M, Onisor C, Rebegea L, Vesa D (2022). Nasopharyngeal carcinoma: A new synthesis of literature data (Review). Exp Ther Med.

[5] Feng Y, Dai Z, Yan R, Li F, Zhong X, Ye H (2021). Outcomes of Recurrent Nasopharyngeal Carcinoma Patients Treated With Salvage Surgery: A Meta-Analysis. Front Oncol.

[6] Xu M, Yao Y, Chen H, Zhang S, Cao S-M, Zhang Z (2019). Genome sequencing analysis identifies Epstein-Barr virus subtypes associated with high risk of nasopharyngeal carcinoma. Nat Genet.

[7] Chen CJ, Liang KY, Chang YS, Wang YF, Hsieh T, Hsu MM (1990). Multiple risk factors of nasopharyngeal carcinoma: Epstein-Barr virus, malarial infection, cigarette smoking and familial tendency. Anticancer Res.

[8] Chang ET, Ye W, Zeng YX, Adami HO (2021). The Evolving Epidemiology of Nasopharyngeal Carcinoma. Cancer Epidemiol Biomarkers Prev.

[9] Okekpa SI, RB SMNM, Mangantig E, Azmi NSA, Zahari SNS, Kaur G, et al. Nasopharyngeal Carcinoma (NPC) Risk Factors: A Systematic Review and Meta-Analysis of the Association with Lifestyle, Diets, Socioeconomic and Sociodemographic in Asian Region. Asian Pac J Cancer Prev. 2019;20(11):3505–14. 10.31557/APJCP.2019.20.11.3505 PMC7063023 31759378

[10] Huang T, Ploner A, Chang ET, Liu Q, Cai Y, Zhang Z (2021). Dietary patterns and risk of nasopharyngeal carcinoma: a population-based case-control study in southern China. Am J Clin Nutr.

[11] Guo X, Johnson RC, Deng H, Liao J, Guan L, Nelson GW (2009). Evaluation of nonviral risk factors for nasopharyngeal carcinoma in a high-risk population of Southern China. Int J Cancer.

[12] Marsh GM, Youk AO, Buchanich JM, Cassidy LD, Lucas LJ, Esmen NA (2002). Pharyngeal cancer mortality among chemical plant workers exposed to formaldehyde. Toxicol Ind Health.

[13] Yao K, Qin H, Gong L, Zhang R, Li L. CYP2E1 polymorphisms and nasopharyngeal carcinoma risk: a meta-analysis. Eur Arch Otorhinolaryngol. 2017;274(1):253–9. 10.1007/s00405-016-4236-6 27491320

[14] Zhao Y, Wang Y, Wu X, Wang J, Zhang L, Jia Y (2016). Quantitative assessment of the association between glutathione S-transferase M1 polymorphism and the risk of developing nasopharyngeal cancer. Oncol Lett.

[15] Liao WL, Chan FC, Chang KP, Chang YW, Chen CH, Su WH, et al. Associations between ALDH Genetic Variants, Alcohol Consumption, and the Risk of Nasopharyngeal Carcinoma in an East Asian Population. Genes. 2021;12(10):1547. 10.3390/genes12101547 PMC8535421 34680942

[16] Sahu SK, Chakrabarti S, Roy SD, Baishya N, Reddy RR, Suklabaidya S (2016). Association of p53 codon72 Arg>Pro polymorphism with susceptibility to nasopharyngeal carcinoma: evidence from a case-control study and meta-analysis. Oncogenesis.

[17] Yang J, Li L, Yin X, Wu F, Shen J, Peng Y (2015). The association between gene polymorphisms and risk of nasopharyngeal carcinoma. Med Oncol.

[18] Surova O, Zhivotovsky B (2013). Various modes of cell death induced by DNA damage. Oncogene.

[19] Jiromaru R, Nakagawa T, Yasumatsu R (2022). Advanced Nasopharyngeal Carcinoma: Current and Emerging Treatment Options. Cancer Manage Res.

[20] Xiao WW, Han F, Lu TX, Chen CY, Huang Y, Zhao C (2009). Treatment outcomes after radiotherapy alone for patients with early-stage nasopharyngeal carcinoma. Int J Radiat Oncol Biol Phys.

[21] Fang FM, Chien CY, Tsai WL, Chen HC, Hsu HC, Lui CC, et al. Quality of life and survival outcome for patients with nasopharyngeal carcinoma receiving three-dimensional conformal radiotherapy vs. intensity-modulated radiotherapy-a longitudinal study. Int J Radiat Oncol Biol Phys. 2008;72(2):356–64. 10.1016/j.ijrobp.2007.12.054 18355980

[22] Tseng M, Ho F, Leong YH, Wong LC, Tham IW, Cheo T (2020). Emerging radiotherapy technologies and trends in nasopharyngeal cancer. Cancer Commun.

[23] Chan AT, Teo PM, Leung TW, Leung SF, Lee WY, Yeo W (1995). A prospective randomized study of chemotherapy adjunctive to definitive radiotherapy in advanced nasopharyngeal carcinoma. Int J Radiat Oncol Biol Phys.

[24] Preliminary results of a randomized trial comparing neoadjuvant chemotherapy (cisplatin, epirubicin, bleomycin) plus radiotherapy vs. radiotherapy alone in stage IV(> or = N2, M0) undifferentiated nasopharyngeal carcinoma: a positive effect on progression-free survival. Int J Radiat Oncol Biol Phys. 1996;35(3):463–9. 10.1016/s0360-3016(96)80007-1 8655368

[25] Maas B, Ho C, Hamilton S, Leedy D, Berthelet E (2018). Impact of Neoadjuvant Chemotherapy on the Administration of Concurrent Chemoradiation for Locally Advanced Nasopharyngeal Carcinoma. Cureus.

[26] Chan ATC, Teo PML, Leung TWT, Johnson PJ. The role of chemotherapy in the management of nasopharyngeal carcinoma. Cancer. 1998;82(6):1003-12. 10.1002/(sici)1097-0142(19980315)82:6<1003::aid-cncr1>3.0.co;2-f 9506343

[27] Song CH, Wu HG, Heo DS, Kim KH, Sung MW, Park CI (2008). Treatment outcomes for radiotherapy alone are comparable with neoadjuvant chemotherapy followed by radiotherapy in early-stage nasopharyngeal carcinoma. Laryngoscope.

[28] Baujat B, Audry H, Bourhis J, Chan AT, Onat H, Chua DT (2006). Chemotherapy in locally advanced nasopharyngeal carcinoma: an individual patient data meta-analysis of eight randomized trials and 1753 patients. Int J Radiat Oncol Biol Phys.

[29] Wang Q, Xu G, Xia Y, Zuo J, Zeng G, Xue Z (2020). Comparison of induction chemotherapy plus concurrent chemoradiotherapy and induction chemotherapy plus radiotherapy in locally advanced nasopharyngeal carcinoma. Oral Oncol.

[30] Mainou BA, Everly DN, Raab-Traub N (2005). Epstein-Barr virus latent membrane protein 1 CTAR1 mediates rodent and human fibroblast transformation through activation of PI3K. Oncogene.

[31] Fendri A, Khabir A, Mnejja W, Sellami-Boudawara T, Daoud J, Frikha M (2009). PIK3CA amplification is predictive of poor prognosis in Tunisian patients with nasopharyngeal carcinoma. Cancer Sci.

[32] Jiang H, Fan D, Zhou G, Li X, Deng H (2010). Phosphatidylinositol 3-kinase inhibitor(LY294002) induces apoptosis of human nasopharyngeal carcinoma in vitro and in vivo. J Exp Clin Cancer Res.

[33] Plotnikov A, Zehorai E, Procaccia S, Seger R (2011). The MAPK cascades: signaling components, nuclear roles and mechanisms of nuclear translocation. Biochem Biophys Acta.

[34] Koul HK, Pal M, Koul S (2013). Role of p38 MAP Kinase Signal Transduction in Solid Tumors. Genes Cancer.

[35] Zhang YJ, Zhang MF, Zhou HF, Yang J (2018). Activation of c-Jun/JNK signaling predicts poor prognosis in nasopharyngeal carcinoma. Int J Clin Exp Pathol.

[36] Tulalamba W, Janvilisri T (2012). Nasopharyngeal carcinoma signaling pathway: an update on molecular biomarkers. Int J Cell Biol.

[37] Yoshizuka N, Chen RM, Xu Z, Liao R, Hong L, Hu WY (2012). A novel function of p38-regulated/activated kinase in endothelial cell migration and tumor angiogenesis. Mol Cell Biol.

[38] Xu J, Ying Y, Xiong G, Lai L, Wang Q, Yang Y (2019). Amyloid β precursor protein silencing attenuates epithelial-mesenchymal transition of nasopharyngeal carcinoma cells via inhibition of the MAPK pathway. Mol Med Rep.

[39] Lin ML, Lu YC, Chung JG, Wang SG, Lin HT, Kang SE (2010). Down-regulation of MMP-2 through the p38 MAPK-NF-kappaB-dependent pathway by aloe-emodin leads to inhibition of nasopharyngeal carcinoma cell invasion. Mol Carcinog.

[40] Hsiao YW, Li CF, Chi JY, Tseng JT, Chang Y, Hsu LJ, et al. CCAAT/enhancer binding protein δ in macrophages contributes to immunosuppression and inhibits phagocytosis in nasopharyngeal carcinoma. Sci Signal. 2013;6(284):ra59. 10.1126/scisignal.2003648 23861541

[41] Hayden MS, Ghosh S (2012). NF-κB, the first quarter-century: remarkable progress and outstanding questions. Genes Dev.

[42] Yi M, Cai J, Li J, Chen S, Zeng Z, Peng Q (2018). Rediscovery of NF-κB signaling in nasopharyngeal carcinoma: How genetic defects of NF-κB pathway interplay with EBV in driving oncogenesis?. J Cell Physiol.

[43] Hui EP, Chan AT, Pezzella F, Turley H, To KF, Poon TC (2002). Coexpression of hypoxia-inducible factors 1alpha and 2alpha, carbonic anhydrase IX, and vascular endothelial growth factor in nasopharyngeal carcinoma and relationship to survival. Clin Cancer Res.

[44] Elser C, Siu LL, Winquist E, Agulnik M, Pond GR, Chin SF (2007). Phase II trial of sorafenib in patients with recurrent or metastatic squamous cell carcinoma of the head and neck or nasopharyngeal carcinoma. J Clin Oncol.

[45] Xue C, Huang Y, Huang PY, Yu QT, Pan JJ, Liu LZ (2013). Phase II study of sorafenib in combination with cisplatin and 5-fluorouracil to treat recurrent or metastatic nasopharyngeal carcinoma. Ann Oncol.

[46] Jiang W, Liang J, Pan Y, Ruan X, Cai R, He Z, et al. Apatinib for locoregionally recurrent or metastatic nasopharyngeal carcinoma after failure of first-line chemotherapy: A multicenter, phase II trial. J Clin Oncol. 2019;37(15_suppl):6030.

[47] Ma B, Hui EP, King A, To KF, Mo F, Leung SF (2008). A phase II study of patients with metastatic or locoregionally recurrent nasopharyngeal carcinoma and evaluation of plasma Epstein-Barr virus DNA as a biomarker of efficacy. Cancer Chemother Pharmacol.

[48] Chua DT, Wei WI, Wong MP, Sham JS, Nicholls J, Au GK (2008). Phase II study of gefitinib for the treatment of recurrent and metastatic nasopharyngeal carcinoma. Head Neck.

[49] You B, Le Tourneau C, Chen EX, Wang L, Jarvi A, Bharadwaj RR (2012). A Phase II trial of erlotinib as maintenance treatment after gemcitabine plus platinum-based chemotherapy in patients with recurrent and/or metastatic nasopharyngeal carcinoma. Am J Clin Oncol.

[50] Hu W, Wang W, Yang P, Zhou C, Yang W, Wu B (2015). Phase I study of icotinib, an EGFR tyrosine kinase inhibitor combined with IMRT in nasopharyngeal carcinoma. Int J Clin Exp Med.

[51] Xu JY, Wei XL, Wang YQ, Wang FH (2022). Current status and advances of immunotherapy in nasopharyngeal carcinoma. Ther Adv Med Oncol.

[52] Su ZY, Siak PY, Leong CO, Cheah SC (2023). The role of Epstein-Barr virus in nasopharyngeal carcinoma. Front Microbiol.

[53] Dasari V, Sinha D, Neller MA, Smith C, Khanna R (2019). Prophylactic and therapeutic strategies for Epstein-Barr virus-associated diseases: emerging strategies for clinical development. Expert Rev Vaccines.

[54] Balfour HH, Schmeling DO, Grimm-Geris JM (2020). The promise of a prophylactic Epstein-Barr virus vaccine. Pediatr Res.

[55] Icheva V, Kayser S, Wolff D, Tuve S, Kyzirakos C, Bethge W (2013). Adoptive transfer of epstein-barr virus (EBV) nuclear antigen 1-specific t cells as treatment for EBV reactivation and lymphoproliferative disorders after allogeneic stem-cell transplantation. J Clin Oncol.

[56] Duraiswamy J, Sherritt M, Thomson S, Tellam J, Cooper L, Connolly G (2003). Therapeutic LMP1 polyepitope vaccine for EBV-associated Hodgkin disease and nasopharyngeal carcinoma. Blood.

[57] Hui EP, Taylor GS, Jia H, Ma BB, Chan SL, Ho R (2013). Phase I trial of recombinant modified vaccinia ankara encoding Epstein-Barr viral tumor antigens in nasopharyngeal carcinoma patients. Can Res.

[58] Smith C, Tsang J, Beagley L, Chua D, Lee V, Li V (2012). Effective treatment of metastatic forms of Epstein-Barr virus-associated nasopharyngeal carcinoma with a novel adenovirus-based adoptive immunotherapy. Can Res.

[59] Chen CY, Lin YS, Chen CH, Chen YJ (2018). Annexin A2-mediated cancer progression and therapeutic resistance in nasopharyngeal carcinoma. J Biomed Sci.

[60] Li J, Chen QY, He J, Li ZL, Tang XF, Chen SP (2015). Phase I trial of adoptively transferred tumor-infiltrating lymphocyte immunotherapy following concurrent chemoradiotherapy in patients with locoregionally advanced nasopharyngeal carcinoma. Oncoimmunology.

[61] Larkin J, Chiarion-Sileni V, Gonzalez R, Grob JJ, Rutkowski P, Lao CD (2019). Five-Year Survival with Combined Nivolumab and Ipilimumab in Advanced Melanoma. N Engl J Med.

[62] Wang S, Chen S, Zhong Q, Liu Y (2023). Immunotherapy for the treatment of advanced nasopharyngeal carcinoma: a promising new era. J Cancer Res Clin Oncol.

[63] Pang X, Huang Z, Zhong T, Zhang P, Wang ZM, Xia M, et al. Cadonilimab, a tetravalent PD-1/CTLA-4 bispecific antibody with trans-binding and enhanced target binding avidity. mAbs. 2023;15(1):2180794. 10.1080/19420862.2023.2180794 PMC10012886 36872527

[64] Nomi T, Sho M, Akahori T, Hamada K, Kubo A, Kanehiro H (2007). Clinical significance and therapeutic potential of the programmed death-1 ligand/programmed death-1 pathway in human pancreatic cancer. Clin Cancer Res.

[65] Ito N, Tsujimoto H, Horiguchi H, Shimazaki H, Miyazaki H, Saitoh D (2020). Clinical Significance of Programmed Death Ligand-1 Expression in Esophageal Squamous Cell Carcinoma. J Surg Res.

[66] Mai H-Q, Chen Q-Y, Chen D, Hu C, Yang K, Wen J (2021). Toripalimab or placebo plus chemotherapy as first-line treatment in advanced nasopharyngeal carcinoma: a multicenter randomized phase 3 trial. Nat Med.

[67] Paget S. The distribution of secondary growths in cancer of the breast. 1889. Cancer Metastasis Rev. 1989;8(2):98–101. 2673568

[68] Mosialos G, Birkenbach M, Yalamanchili R, VanArsdale T, Ware C, Kieff E (1995). The Epstein-Barr virus transforming protein LMP1 engages signaling proteins for the tumor necrosis factor receptor family. Cell.

[69] Dawson CW, Port RJ, Young LS (2012). The role of the EBV-encoded latent membrane proteins LMP1 and LMP2 in the pathogenesis of nasopharyngeal carcinoma (NPC). Semin Cancer Biol.

[70] Leight ER, Sugden B (2000). EBNA-1: a protein pivotal to latent infection by Epstein-Barr virus. Rev Med Virol.

[71] Forder A, Stewart GL, Telkar N, Lam WL, Garnis C (2022). New insights into the tumour immune microenvironment of nasopharyngeal carcinoma. Curr Res Immunol.

[72] Bruce JP, To KF, Lui VWY, Chung GTY, Chan YY, Tsang CM (2021). Whole-genome profiling of nasopharyngeal carcinoma reveals viral-host co-operation in inflammatory NF-κB activation and immune escape. Nat Commun.

[73] Li YY, Chung GT, Lui VW, To KF, Ma BB, Chow C (2017). Exome and genome sequencing of nasopharynx cancer identifies NF-κB pathway activating mutations. Nat Commun.

[74] Wang C, Jiang S, Ke L, Zhang L, Li D, Liang J (2019). Genome-wide CRISPR-based gene knockout screens reveal cellular factors and pathways essential for nasopharyngeal carcinoma. J Biol Chem.

[75] Lo AK, Dawson CW, Lung HL, Wong KL, Young LS (2021). The Role of EBV-Encoded LMP1 in the NPC Tumor Microenvironment: From Function to Therapy. Front Oncol.

[76] Zhao J, Guo C, Xiong F, Yu J, Ge J, Wang H (2020). Single cell RNA-seq reveals the landscape of tumor and infiltrating immune cells in nasopharyngeal carcinoma. Cancer Lett.

[77] Ooft ML, van Ipenburg JA, Sanders ME, Kranendonk M, Hofland I, de Bree R (2018). Prognostic role of tumour-associated macrophages and regulatory T cells in EBV-positive and EBV-negative nasopharyngeal carcinoma. J Clin Pathol.

[78] Yang D, Guo P, He T, Powell CA (2021). Role of endothelial cells in tumor microenvironment. Clin Transl Med.

[79] Aird WC (2012). Endothelial cell heterogeneity. Cold Spring Harb Perspect Med.

[80] Murono S, Inoue H, Tanabe T, Joab I, Yoshizaki T, Furukawa M (2001). Induction of cyclooxygenase-2 by Epstein-Barr virus latent membrane protein 1 is involved in vascular endothelial growth factor production in nasopharyngeal carcinoma cells. Proc Natl Acad Sci USA.

[81] Farina A, Rosato E, York M, Gewurz BE, Trojanowska M, Farina GA (2021). Innate Immune Modulation Induced by EBV Lytic Infection Promotes Endothelial Cell Inflammation and Vascular Injury in Scleroderma. Front Immunol.

[82] Cheng H-W, Chen Y-F, Wong J-M, Weng C-W, Chen H-Y, Yu S-L (2017). Cancer cells increase endothelial cell tube formation and survival by activating the PI3K/Akt signalling pathway. J Exp Clin Cancer Res.

[83] Maishi N, Hida K (2017). Tumor endothelial cells accelerate tumor metastasis. Cancer Sci.

[84] Yadav A, Kumar B, Yu JG, Old M, Teknos TN, Kumar P (2015). Tumor-Associated Endothelial Cells Promote Tumor Metastasis by Chaperoning Circulating Tumor Cells and Protecting Them from Anoikis. PLoS ONE.

[85] Kalluri R, Zeisberg M (2006). Fibroblasts in cancer. Nat Rev Cancer.

[86] Wu X, Zhou Z, Xu S, Liao C, Chen X, Li B (2020). Extracellular vesicle packaged LMP1-activated fibroblasts promote tumor progression via autophagy and stroma-tumor metabolism coupling. Cancer Lett.

[87] Davis AM, Rapley A, Dawson CW, Young LS, Morris MA. The EBV-Encoded Oncoprotein, LMP1, Recruits and Transforms Fibroblasts via an ERK-MAPK-Dependent Mechanism. Pathogens. 2021;10(8):982. 10.3390/pathogens10080982 PMC8400670 34451446

[88] De Palma M, Biziato D, Petrova TV (2017). Microenvironmental regulation of tumour angiogenesis. Nat Rev Cancer.

[89] Crawford Y, Kasman I, Yu L, Zhong C, Wu X, Modrusan Z (2009). PDGF-C mediates the angiogenic and tumorigenic properties of fibroblasts associated with tumors refractory to anti-VEGF treatment. Cancer Cell.

[90] Orimo A, Gupta PB, Sgroi DC, Arenzana-Seisdedos F, Delaunay T, Naeem R (2005). Stromal fibroblasts present in invasive human breast carcinomas promote tumor growth and angiogenesis through elevated SDF-1/CXCL12 secretion. Cell.

[91] Huang W, Zhang L, Yang M, Wu X, Wang X, Huang W (2021). Cancer-associated fibroblasts promote the survival of irradiated nasopharyngeal carcinoma cells via the NF-κB pathway. J Exp Clin Cancer Res.

[92] Galli F, Aguilera JV, Palermo B, Markovic SN, Nisticò P, Signore A (2020). Relevance of immune cell and tumor microenvironment imaging in the new era of immunotherapy. J Exp Clin Cancer Res.

[93] Bruni D, Angell HK, Galon J (2020). The immune contexture and Immunoscore in cancer prognosis and therapeutic efficacy. Nat Rev Cancer.

[94] Chang SH (2019). T helper 17 (Th17) cells and interleukin-17 (IL-17) in cancer. Arch Pharmacal Res.

[95] Ressing ME, van Gent M, Gram AM, Hooykaas MJ, Piersma SJ, Wiertz EJ (2015). Immune Evasion by Epstein-Barr Virus. Curr Top Microbiol Immunol.

[96] Chen M, Jin F, Ma L (2018). The detection and significance of T cells in nasopharyngeal carcinoma patients. J Cancer Res Ther.

[97] Chan OS, Kowanetz M, Ng WT, Koeppen H, Chan LK, Yeung RM (2017). Characterization of PD-L1 expression and immune cell infiltration in nasopharyngeal cancer. Oral Oncol.

[98] Yang L, Liu G, Li Y, Pan Y (2022). The emergence of tumor-infiltrating lymphocytes in nasopharyngeal carcinoma: Predictive value and immunotherapy implications. Genes Dis.

[99] Ohue Y, Nishikawa H. Regulatory T (Treg) cells in cancer: Can Treg cells be a new therapeutic target? Cancer Sci. 2019;110(7):2080–9. 10.1111/cas.14069 PMC6609813 31102428

[100] Iglesia MD, Parker JS, Hoadley KA, Serody JS, Perou CM, Vincent BG. Genomic Analysis of Immune Cell Infiltrates Across 11 Tumor Types. J Natl Cancer Inst. 2016;108(11):djw144. 10.1093/jnci/djw144 PMC5241901 27335052

[101] Li S, Huang C, Hu G, Ma J, Chen Y, Zhang J (2020). Tumor-educated B cells promote renal cancer metastasis via inducing the IL-1β/HIF-2α/Notch1 signals. Cell Death Dis.

[102] Gong L, Kwong DL, Dai W, Wu P, Li S, Yan Q (2021). Comprehensive single-cell sequencing reveals the stromal dynamics and tumor-specific characteristics in the microenvironment of nasopharyngeal carcinoma. Nat Commun.

[103] Chung MKY, Gong L, Kwong DL, Lee VH, Lee AW, Guan XY (2023). Functions of double-negative B cells in autoimmune diseases, infections, and cancers. EMBO Mol Med.

[104] Tsai CY, Sakakibara S, Yasui T, Minamitani T, Okuzaki D, Kikutani H (2018). Bystander inhibition of humoral immune responses by Epstein-Barr virus LMP1. Int Immunol.

[105] Anastasiadou E, Garg N, Bigi R, Yadav S, Campese AF, Lapenta C (2015). Epstein-Barr virus infection induces miR-21 in terminally differentiated malignant B cells. Int J Cancer.

[106] Vivier E, Raulet DH, Moretta A, Caligiuri MA, Zitvogel L, Lanier LL (2011). Innate or adaptive immunity? The example of natural killer cells. Science.

[107] Makowska A, Braunschweig T, Denecke B, Shen L, Baloche V, Busson P (2019). Interferon β and Anti-PD-1/PD-L1 Checkpoint Blockade Cooperate in NK Cell-Mediated Killing of Nasopharyngeal Carcinoma Cells. Transl Oncol.

[108] Glasner A, Levi A, Enk J, Isaacson B, Viukov S, Orlanski S (2018). NKp46 Receptor-Mediated Interferon-γ Production by Natural Killer Cells Increases Fibronectin 1 to Alter Tumor Architecture and Control Metastasis. Immunity.

[109] Ghiringhelli F, Ménard C, Terme M, Flament C, Taieb J, Chaput N (2005). CD4+CD25+ regulatory T cells inhibit natural killer cell functions in a transforming growth factor-beta-dependent manner. J Exp Med.

[110] Melaiu O, Lucarini V, Cifaldi L, Fruci D (2019). Influence of the Tumor Microenvironment on NK Cell Function in Solid Tumors. Front Immunol.

[111] Lo KW, To KF, Huang DP (2004). Focus on nasopharyngeal carcinoma. Cancer Cell.

[112] Everett H, McFadden G (2001). Viruses and apoptosis: meddling with mitochondria. Virology.

[113] Yoshimori M, Imadome K, Komatsu H, Wang L, Saitoh Y, Yamaoka S (2014). CD137 expression is induced by Epstein-Barr virus infection through LMP1 in T or NK cells and mediates survival promoting signals. PLoS ONE.

[114] Evans R, Alexander P (1970). Cooperation of Immune Lymphoid Cells with Macrophages in Tumour Immunity. Nature.

[115] Mantovani A (1978). Effects on in vitro tumor growth of murine macrophages isolated from sarcoma lines differing in immunogenicity and metastasizing capacity. Int J Cancer.

[116] Murray PJ, Allen JE, Biswas SK, Fisher EA, Gilroy DW, Goerdt S (2014). Macrophage activation and polarization: nomenclature and experimental guidelines. Immunity.

[117] Noy R, Pollard JW (2014). Tumor-associated macrophages: from mechanisms to therapy. Immunity.

[118] Wang Y, Sun Q, Ye Y, Sun X, Xie S, Zhan Y, et al. FGF-2 signaling in nasopharyngeal carcinoma modulates pericyte-macrophage crosstalk and metastasis. JCI insight. 2022;7(10):e157874. 10.1172/jci.insight.157874 PMC9220856 35439170

[119] Sun X, Ingman WV (2014). Cytokine networks that mediate epithelial cell-macrophage crosstalk in the mammary gland: implications for development and cancer. J Mammary Gland Biol Neoplasia.

[120] Jin S, Li R, Chen MY, Yu C, Tang LQ, Liu YM (2020). Single-cell transcriptomic analysis defines the interplay between tumor cells, viral infection, and the microenvironment in nasopharyngeal carcinoma. Cell Res.

[121] Egeblad M, Rasch MG, Weaver VM (2010). Dynamic interplay between the collagen scaffold and tumor evolution. Curr Opin Cell Biol.

[122] Bonnans C, Chou J, Werb Z (2014). Remodelling the extracellular matrix in development and disease. Nat Rev Mol Cell Biol.

[123] Shimizu Y, Shaw S (1991). Lymphocyte interactions with extracellular matrix. FASEB J.

[124] Liu S-X, Zhao G-X, Lin R-B, Zeng M-S, Zhong QJAoNC. Classifying the tumor microenvironment to stratify nasopharyngeal carcinoma patients. Ann Nasopharynx Cancer. 2022;6:8.

[125] Chang CH, Qiu J, O'Sullivan D, Buck MD, Noguchi T, Curtis JD (2015). Metabolic Competition in the Tumor Microenvironment Is a Driver of Cancer Progression. Cell.

[126] Chang KP, Chang YT, Wu CC, Liu YL, Chen MC, Tsang NM (2011). Multiplexed immunobead-based profiling of cytokine markers for detection of nasopharyngeal carcinoma and prognosis of patient survival. Head Neck.

[127] Sun W, Liu DB, Li WW, Zhang LL, Long GX, Wang JF (2014). Interleukin-6 promotes the migration and invasion of nasopharyngeal carcinoma cell lines and upregulates the expression of MMP-2 and MMP-9. Int J Oncol.

[128] Ben Nasr H, Chahed K, Mestiri S, Bouaouina N, Snoussi K, Chouchane L (2007). Association of IL-8 (-251)T/A polymorphism with susceptibility to and aggressiveness of nasopharyngeal carcinoma. Hum Immunol.

[129] Wei YS, Lan Y, Tang RG, Xu QQ, Huang Y, Nong HB (2007). Single nucleotide polymorphism and haplotype association of the interleukin-8 gene with nasopharyngeal carcinoma. Clin Immunol.

[130] Huang CY, Chang WS, Tsai CW, Hsia TC, Shen TC, Bau DT (2018). The contribution of interleukin-8 genotypes and expression to nasopharyngeal cancer susceptibility in Taiwan. Medicine.

[131] Katanov C, Lerrer S, Liubomirski Y, Leider-Trejo L, Meshel T, Bar J (2015). Regulation of the inflammatory profile of stromal cells in human breast cancer: prominent roles for TNF-α and the NF-κB pathway. Stem Cell Res Ther.

[132] Yu Y, Ke L, Xia WX, Xiang Y, Lv X, Bu J (2019). Elevated Levels of TNF-α and Decreased Levels of CD68-Positive Macrophages in Primary Tumor Tissues Are Unfavorable for the Survival of Patients With Nasopharyngeal Carcinoma. Technol Cancer Res Treat.

[133] Hu H, Tang KF, Chua YN, Lu J, Feng P, Chew CT (2004). Expression of interleukin-18 by nasopharyngeal carcinoma cells: a factor that possibly initiates the massive leukocyte infiltration. Hum Pathol.

[134] Liou AK, Soon G, Tan L, Peng Y, Cher BM, Goh BC (2020). Elevated IL18 levels in Nasopharyngeal carcinoma induced PD-1 expression on NK cells in TILS leading to poor prognosis. Oral Oncol.

[135] Lo AK, Dawson CW, Young LS, Ko CW, Hau PM, Lo KW (2015). Activation of the FGFR1 signalling pathway by the Epstein-Barr virus-encoded LMP1 promotes aerobic glycolysis and transformation of human nasopharyngeal epithelial cells. J Pathol.

[136] Ho PC, Bihuniak JD, Macintyre AN, Staron M, Liu X, Amezquita R (2015). Phosphoenolpyruvate Is a Metabolic Checkpoint of Anti-tumor T Cell Responses. Cell.

[137] Cascone T, McKenzie JA, Mbofung RM, Punt S, Wang Z, Xu C (2018). Increased Tumor Glycolysis Characterizes Immune Resistance to Adoptive T Cell Therapy. Cell Metab.

[138] Gong T, Zheng C, Ou X, Zheng J, Yu J, Chen S (2022). Glutamine metabolism in cancers: Targeting the oxidative homeostasis. Front Oncol.

[139] Ma G, Zhang Z, Li P, Zhang Z, Zeng M, Liang Z (2022). Reprogramming of glutamine metabolism and its impact on immune response in the tumor microenvironment. Cell Commun Signal.

[140] Fischer K, Hoffmann P, Voelkl S, Meidenbauer N, Ammer J, Edinger M (2007). Inhibitory effect of tumor cell-derived lactic acid on human T cells. Blood.

[141] Goetze K, Walenta S, Ksiazkiewicz M, Kunz-Schughart LA, Mueller-Klieser W (2011). Lactate enhances motility of tumor cells and inhibits monocyte migration and cytokine release. Int J Oncol.

[142] Colegio OR, Chu NQ, Szabo AL, Chu T, Rhebergen AM, Jairam V (2014). Functional polarization of tumour-associated macrophages by tumour-derived lactic acid. Nature.

[143] Moon YW, Hajjar J, Hwu P, Naing A (2015). Targeting the indoleamine 2,3-dioxygenase pathway in cancer. J Immunother Cancer.

[144] Munn DH, Shafizadeh E, Attwood JT, Bondarev I, Pashine A, Mellor AL (1999). Inhibition of T cell proliferation by macrophage tryptophan catabolism. J Exp Med.

[145] Campesato LF, Budhu S, Tchaicha J, Weng CH, Gigoux M, Cohen IJ (2020). Blockade of the AHR restricts a Treg-macrophage suppressive axis induced by L-Kynurenine. Nat Commun.

[146] Chen QY, Gao B, Tong D, Huang C (2023). Crosstalk between extracellular vesicles and tumor-associated macrophage in the tumor microenvironment. Cancer Lett.

[147] Gu M, Li L, Zhang Z, Chen J, Zhang W, Zhang J (2017). PFKFB3 promotes proliferation, migration and angiogenesis in nasopharyngeal carcinoma. J Cancer.

[148] Li F, Zhao X, Sun R, Ou J, Huang J, Yang N (2020). EGFR-rich extracellular vesicles derived from highly metastatic nasopharyngeal carcinoma cells accelerate tumour metastasis through PI3K/AKT pathway-suppressed ROS. J Extracell Vesicles.

[149] Meckes DG, Shair KH, Marquitz AR, Kung CP, Edwards RH, Raab-Traub N (2010). Human tumor virus utilizes exosomes for intercellular communication. Proc Natl Acad Sci USA.

[150] Liao C, Zhou Q, Zhang Z, Wu X, Zhou Z, Li B (2020). Epstein-Barr virus-encoded latent membrane protein 1 promotes extracellular vesicle secretion through syndecan-2 and synaptotagmin-like-4 in nasopharyngeal carcinoma cells. Cancer Sci.

[151] Zuo L, Xie Y, Tang J, Xin S, Liu L, Zhang S (2019). Targeting Exosomal EBV-LMP1 Transfer and miR-203 Expression via the NF-κB Pathway: The Therapeutic Role of Aspirin in NPC. Mol Ther Nucleic Acids.

[152] Ikeda M, Longnecker R (2007). Cholesterol is critical for Epstein-Barr virus latent membrane protein 2A trafficking and protein stability. Virology.

[153] Wang J, Liu Y, Zhang Y, Li X, Fang M, Qian D. Targeting exosomes enveloped EBV-miR-BART1–5p-antagomiRs for NPC therapy through both anti-vasculogenic mimicry and anti-angiogenesis. Cancer Med. 2023;12(11):12608–21. 10.1002/cam4.5941 PMC10278492 37097161

[154] Li D-K, Chen X-R, Wang L-N, Wang J-H, Li J-K, Zhou Z-Y (2022). Exosomal HMGA2 protein from EBV-positive NPC cells destroys vascular endothelial barriers and induces endothelial-to-mesenchymal transition to promote metastasis. Cancer Gene Ther.

[155] Shi S, Zhang Q, Xia Y, You B, Shan Y, Bao L (2016). Mesenchymal stem cell-derived exosomes facilitate nasopharyngeal carcinoma progression. Am J Cancer Res.

[156] Wan FZ, Chen KH, Sun YC, Chen XC, Liang RB, Chen L (2020). Exosomes overexpressing miR-34c inhibit malignant behavior and reverse the radioresistance of nasopharyngeal carcinoma. J Transl Med.

[157] Kachalaki S, Ebrahimi M, Mohamed Khosroshahi L, Mohammadinejad S, Baradaran B (2016). Cancer chemoresistance; biochemical and molecular aspects: a brief overview. Eur J Pharm Sci.

[158] Young LS, Rickinson AB (2004). Epstein-Barr virus: 40 years on. Nat Rev Cancer.

[159] Kim JH, Kim WS, Yun Y, Park C (2010). Epstein-Barr virus latent membrane protein 1 increases chemo-resistance of cancer cells via cytoplasmic sequestration of Pim-1. Cell Signal.

[160] Li SS, Yang S, Wang S, Yang XM, Tang QL, Wang SH (2011). Latent membrane protein 1 mediates the resistance of nasopharyngeal carcinoma cells to TRAIL-induced apoptosis by activation of the PI3K/Akt signaling pathway. Oncol Rep.

[161] Yang GD, Huang TJ, Peng LX, Yang CF, Liu RY, Huang HB (2013). Epstein-Barr Virus_Encoded LMP1 upregulates microRNA-21 to promote the resistance of nasopharyngeal carcinoma cells to cisplatin-induced Apoptosis by suppressing PDCD4 and Fas-L. PLoS ONE.

[162] Yang CF, Peng LX, Huang TJ, Yang GD, Chu QQ, Liang YY (2014). Cancer stem-like cell characteristics induced by EB virus-encoded LMP1 contribute to radioresistance in nasopharyngeal carcinoma by suppressing the p53-mediated apoptosis pathway. Cancer Lett.

[163] Lu J, Tang M, Li H, Xu Z, Weng X, Li J (2016). EBV-LMP1 suppresses the DNA damage response through DNA-PK/AMPK signaling to promote radioresistance in nasopharyngeal carcinoma. Cancer Lett.

[164] Wu Q, Han T, Sheng X, Zhang N, Wang P (2018). Downregulation of EB virus miR-BART4 inhibits proliferation and aggressiveness while promoting radiosensitivity of nasopharyngeal carcinoma. Biomed Pharmacother.

[165] Gao W, Li ZH, Chen S, Chan JY, Yin M, Zhang MJ (2017). Epstein-Barr virus encoded microRNA BART7 regulates radiation sensitivity of nasopharyngeal carcinoma. Oncotarget.

[166] Zhou X, Zheng J, Tang Y, Lin Y, Wang L, Li Y, et al. EBV encoded miRNA BART8–3p promotes radioresistance in nasopharyngeal carcinoma by regulating ATM/ATR signaling pathway. Biosci Rep. 2019;39(9):BSR20190415. 10.1042/BSR20190415 PMC6744588 31471531

[167] Liu Y, Jiang Q, Liu X, Lin X, Tang Z, Liu C (2019). Cinobufotalin powerfully reversed EBV-miR-BART22-induced cisplatin resistance via stimulating MAP2K4 to antagonize non-muscle myosin heavy chain IIA/glycogen synthase 3beta/beta-catenin signaling pathway. EBioMedicine.

[168] Cai LM, Lyu XM, Luo WR, Cui XF, Ye YF, Yuan CC (2015). EBV-miR-BART7-3p promotes the EMT and metastasis of nasopharyngeal carcinoma cells by suppressing the tumor suppressor PTEN. Oncogene.

[169] Cai L, Long Y, Chong T, Cai W, Tsang CM, Zhou X (2019). EBV-miR-BART7-3p Imposes Stemness in Nasopharyngeal Carcinoma Cells by Suppressing SMAD7. Front Genet.

[170] Choy EY, Siu KL, Kok KH, Lung RW, Tsang CM, To KF (2008). An Epstein-Barr virus-encoded microRNA targets PUMA to promote host cell survival. J Exp Med.

[171] Dasari S, Tchounwou PB (2014). Cisplatin in cancer therapy: molecular mechanisms of action. Eur J Pharmacol.

[172] Werden SJ, Sphyris N, Sarkar TR, Paranjape AN, LaBaff AM, Taube JH (2016). Phosphorylation of serine 367 of FOXC2 by p38 regulates ZEB1 and breast cancer metastasis, without impacting primary tumor growth. Oncogene.

[173] Liao GB, Li XZ, Zeng S, Liu C, Yang SM, Yang L (2018). Regulation of the master regulator FOXM1 in cancer. Cell Commun Signal.

[174] Hou Y, Zhu Q, Li Z, Peng Y, Yu X, Yuan B (2017). The FOXM1-ABCC5 axis contributes to paclitaxel resistance in nasopharyngeal carcinoma cells. Cell Death Dis.

[175] Zhou Z, Zhang L, Xie B, Wang X, Yang X, Ding N (2015). FOXC2 promotes chemoresistance in nasopharyngeal carcinomas via induction of epithelial mesenchymal transition. Cancer Lett.

[176] Li D, Ye L, Lei Y, Wan J, Chen H (2019). Downregulation of FoxM1 sensitizes nasopharyngeal carcinoma cells to cisplatin via inhibition of MRN-ATM-mediated DNA repair. BMB Rep.

[177] Hong X, Liu N, Liang Y, He Q, Yang X, Lei Y (2020). Circular RNA CRIM1 functions as a ceRNA to promote nasopharyngeal carcinoma metastasis and docetaxel chemoresistance through upregulating FOXQ1. Mol Cancer.

[178] Guo X, Gao C, Yang D-H, Li S (2023). Exosomal circular RNAs: A chief culprit in cancer chemotherapy resistance. Drug Resist Updates.

[179] Yu L-L, Xiao Q, Yu B, Lv Q-L, Liu Z-Q, Yin J-Y (2023). CircRNAs in tumor immunity and immunotherapy: Perspectives from innate and adaptive immunity. Cancer Lett.

[180] Fan J, To KKW, Chen Z-S, Fu L (2023). ABC transporters affects tumor immune microenvironment to regulate cancer immunotherapy and multidrug resistance. Drug Resist Updates.

[181] Wang J-Q, Wu Z-X, Yang Y, Teng Q-X, Li Y-D, Lei Z-N, et al. ATP-binding cassette (ABC) transporters in cancer: A review of recent updates. J Evid Based Med. 2021;14(3):232–56. 10.1111/jebm.12434 34388310

[182] Larbcharoensub N, Leopairat J, Sirachainan E, Narkwong L, Bhongmakapat T, Rasmeepaisarn K (2008). Association between multidrug resistance-associated protein 1 and poor prognosis in patients with nasopharyngeal carcinoma treated with radiotherapy and concurrent chemotherapy. Hum Pathol.

[183] Wu ZX, Teng QX, Yang Y, Acharekar N, Wang JQ, He M (2022). MET inhibitor tepotinib antagonizes multidrug resistance mediated by ABCG2 transporter: In vitro and in vivo study. Acta Pharmaceutica Sinica B.

[184] Najafi M, Farhood B, Mortezaee K (2019). Cancer stem cells (CSCs) in cancer progression and therapy. J Cell Physiol.

[185] Nunes T, Hamdan D, Leboeuf C, El Bouchtaoui M, Gapihan G, Nguyen TT, et al. Targeting Cancer Stem Cells to Overcome Chemoresistance. Int J Mol Sci. 2018;19(12):4036. 10.3390/ijms19124036 PMC6321478 30551640

[186] Guan GF, Zhang DJ, Zheng Y, Wen LJ, Yu DJ, Lu YQ (2015). Abnormal Wnt signaling and overexpression of ABCG2 contributes to drug efflux properties of side population cells in nasopharyngeal carcinoma. Mol Med Rep.

[187] Kentsis A, Topisirovic I, Culjkovic B, Shao L, Borden KL (2004). Ribavirin suppresses eIF4E-mediated oncogenic transformation by physical mimicry of the 7-methyl guanosine mRNA cap. Proc Natl Acad Sci U S A.

[188] Sonenberg N, Dever TE (2003). Eukaryotic translation initiation factors and regulators. Curr Opin Struct Biol.

[189] Truitt ML, Conn CS, Shi Z, Pang X, Tokuyasu T, Coady AM (2015). Differential Requirements for eIF4E Dose in Normal Development and Cancer. Cell.

[190] Chen SP, Yang Q, Wang CJ, Zhang LJ, Fang Y, Lei FY (2014). Transducin beta-like 1 X-linked receptor 1 suppresses cisplatin sensitivity in nasopharyngeal carcinoma via activation of NF-kappaB pathway. Mol Cancer.

[191] Clarke MF, Dick JE, Dirks PB, Eaves CJ, Jamieson CH, Jones DL (2006). Cancer stem cells–perspectives on current status and future directions: AACR Workshop on cancer stem cells. Cancer Res.

[192] Gao W, Li JZ, Chen SQ, Chu CY, Chan JY, Wong TS (2017). BEX3 contributes to cisplatin chemoresistance in nasopharyngeal carcinoma. Cancer Med.

[193] Luo W, Li S, Peng B, Ye Y, Deng X, Yao K (2013). Embryonic stem cells markers SOX2, OCT4 and Nanog expression and their correlations with epithelial-mesenchymal transition in nasopharyngeal carcinoma. PLoS ONE.

[194] Olivares-Urbano MA, Griñán-Lisón C, Marchal JA, Núñez MI. CSC Radioresistance: A Therapeutic Challenge to Improve Radiotherapy Effectiveness in Cancer. Cells. 2020;9(7):1651. 10.3390/cells9071651 PMC7407195 32660072

[195] Oshimori N, Oristian D, Fuchs E (2015). TGF-beta promotes heterogeneity and drug resistance in squamous cell carcinoma. Cell.

[196] Bissey PA, Law JH, Bruce JP, Shi W, Renoult A, Chua MLK (2018). Dysregulation of the MiR-449b target TGFBI alters the TGFbeta pathway to induce cisplatin resistance in nasopharyngeal carcinoma. Oncogenesis.

[197] Meldolesi J (2018). Exosomes and Ectosomes in Intercellular Communication. Curr Biol.

[198] Huang L, Hu C, Chao H, Zhang Y, Li Y, Hou J (2019). Drug-resistant endothelial cells facilitate progression, EMT and chemoresistance in nasopharyngeal carcinoma via exosomes. Cell Signal.

[199] Pang K, Shi Z-D, Wei L-Y, Dong Y, Ma Y-Y, Wang W (2023). Research progress of therapeutic effects and drug resistance of immunotherapy based on PD-1/PD-L1 blockade. Drug Resist Updates.

[200] O'Donnell JS, Long GV, Scolyer RA, Teng MW, Smyth MJ (2017). Resistance to PD1/PDL1 checkpoint inhibition. Cancer Treat Rev.

[201] Ma Y, Chen X, Wang A, Zhao H, Lin Q, Bao H, et al. Copy number loss in granzyme genes confers resistance to immune checkpoint inhibitor in nasopharyngeal carcinoma. J Immunother Cancer. 2021;9(3):e002014. 10.1136/jitc-2020-002014 PMC7978327 33737344

[202] Liu WN, Fong SY, Tan WWS, Tan SY, Liu M, Cheng JY, et al. Establishment and Characterization of Humanized Mouse NPC-PDX Model for Testing Immunotherapy. Cancers. 2020;12(4):1025. 10.3390/cancers12041025 PMC7225949 32331230

[203] Kok VC (2020). Current Understanding of the Mechanisms Underlying Immune Evasion From PD-1/PD-L1 Immune Checkpoint Blockade in Head and Neck Cancer. Front Oncol.

[204] Chen L, Diao L, Yang Y, Yi X, Rodriguez BL, Li Y (2018). CD38-Mediated Immunosuppression as a Mechanism of Tumor Cell Escape from PD-1/PD-L1 Blockade. Cancer Discov.

[205] Tang Y, He Y, Shi L, Yang L, Wang J, Lian Y (2017). Co-expression of AFAP1-AS1 and PD-1 predicts poor prognosis in nasopharyngeal carcinoma. Oncotarget.

[206] Kok VC. Current Understanding of the Mechanisms Underlying Immune Evasion From PD-1/PD-L1 Immune Checkpoint Blockade in Head and Neck Cancer. Front Oncol. 2020;10:268. 10.3389/fonc.2020.00268 PMC7058818 32185135

[207] Rahma OE, Hodi FS (2019). The Intersection between Tumor Angiogenesis and Immune Suppression. Clin Cancer Res.

[208] Chao PZ, Hsieh MS, Cheng CW, Hsu TJ, Lin YT, Lai CH (2015). Dendritic cells respond to nasopharygeal carcinoma cells through annexin A2-recognizing DC-SIGN. Oncotarget.

[209] Hopkins R, Xiang W, Marlier D, Au VB, Ching Q, Wu LX (2021). Monocytic Myeloid-Derived Suppressor Cells Underpin Resistance to Adoptive T Cell Therapy in Nasopharyngeal Carcinoma. Mol Ther.

[210] Gabrilovich DI, Nagaraj S (2009). Myeloid-derived suppressor cells as regulators of the immune system. Nat Rev Immunol.

[211] Strand S, Hofmann WJ, Hug H, Müller M, Otto G, Strand D (1996). Lymphocyte apoptosis induced by CD95 (APO-1/Fas) ligand-expressing tumor cells–a mechanism of immune evasion?. Nat Med.

[212] Dotti G, Savoldo B, Pule M, Straathof KC, Biagi E, Yvon E (2005). Human cytotoxic T lymphocytes with reduced sensitivity to Fas-induced apoptosis. Blood.

[213] Guan S, Wei J, Huang L, Wu L (2020). Chemotherapy and chemo-resistance in nasopharyngeal carcinoma. Eur J Med Chem.

[214] Zhu X, Cong J, Lin Z, Sun J, Yang B, Li A (2020). Inhibition of HMGB1 Overcomes Resistance to Radiation and Chemotherapy in Nasopharyngeal Carcinoma. Onco Targets Ther.

[215] Zhu X, Sun L, Wang Y (2016). High mobility group box 1 (HMGB1) is upregulated by the Epstein-Barr virus infection and promotes the proliferation of human nasopharyngeal carcinoma cells. Acta Otolaryngol.

[216] Wang S, Claret FX, Wu W (2019). MicroRNAs as Therapeutic Targets in Nasopharyngeal Carcinoma. Front Oncol.

[217] Zhang JX, Qian D, Wang FW, Liao DZ, Wei JH, Tong ZT (2013). MicroRNA-29c enhances the sensitivities of human nasopharyngeal carcinoma to cisplatin-based chemotherapy and radiotherapy. Cancer Lett.

[218] Sajid A, Rahman H, Ambudkar SV (2023). Advances in the structure, mechanism and targeting of chemoresistance-linked ABC transporters. Nat Rev Cancer.

[219] Engle K, Kumar G (2022). Cancer multidrug-resistance reversal by ABCB1 inhibition: A recent update. Eur J Med Chem.

[220] Sucha S, Sorf A, Svoren M, Vagiannis D, Ahmed F, Visek B, et al. ABCB1 as a potential beneficial target of midostaurin in acute myeloid leukemia. Biomed Pharmacother. 2022;150:112962. 10.1016/j.biopha.2022.112962 35462331

[221] Wu ZX, Teng QX, Cai CY, Wang JQ, Lei ZN, Yang Y (2019). Tepotinib reverses ABCB1-mediated multidrug resistance in cancer cells. Biochem Pharmacol.

[222] Robinson AN, Tebase BG, Francone SC, Huff LM, Kozlowski H, Cossari D (2019). Coexpression of ABCB1 and ABCG2 in a Cell Line Model Reveals Both Independent and Additive Transporter Function. Drug Metab Dispos.

[223] Duan X, Luo M, Li J, Shen Z, Xie K (2022). Overcoming therapeutic resistance to platinum-based drugs by targeting Epithelial-Mesenchymal transition. Front Oncol.

[224] Zhou D, Ye C, Pan Z, Deng Y (2021). SATB1 Knockdown Inhibits Proliferation and Invasion and Decreases Chemoradiation Resistance in Nasopharyngeal Carcinoma Cells by Reversing EMT and Suppressing MMP-9. Int J Med Sci.

[225] Yao Y, Pang T, Cheng Y, Yong W, Kang H, Zhao Y (2020). Positive Correlative over-Expression between eIF4E and Snail in Nasopharyngeal Carcinoma Promotes its Metastasis and Resistance to Cisplatin. Pathol Oncol Res.

[226] Li S, Zhang X, Zhang R, Liang Z, Liao W, Du Z (2017). Hippo pathway contributes to cisplatin resistant-induced EMT in nasopharyngeal carcinoma cells. Cell Cycle.

[227] Li T, Li Y (2023). Quercetin acts as a novel anti-cancer drug to suppress cancer aggressiveness and cisplatin-resistance in nasopharyngeal carcinoma (NPC) through regulating the yes-associated protein/Hippo signaling pathway. Immunobiology.

[228] Bugide S, Gonugunta VK, Penugurti V, Malisetty VL, Vadlamudi RK, Manavathi B (2017). HPIP promotes epithelial-mesenchymal transition and cisplatin resistance in ovarian cancer cells through PI3K/AKT pathway activation. Cell Oncol (Dordr).

[229] Mrkvicova A, Chmelarova M, Peterova E, Havelek R, Baranova I, Kazimirova P (2019). The effect of sodium butyrate and cisplatin on expression of EMT markers. PLoS ONE.

[230] Cheng Y, Mo F, Li Q, Han X, Shi H, Chen S (2021). Targeting CXCR2 inhibits the progression of lung cancer and promotes therapeutic effect of cisplatin. Mol Cancer.

[231] Peng QX, Han YW, Zhang YL, Hu J, Fan J, Fu SZ (2017). Apatinib inhibits VEGFR-2 and angiogenesis in an in vivo murine model of nasopharyngeal carcinoma. Oncotarget.

[232] Chong WQ, Lim CM, Sinha AK, Tan CS, Chan GHJ, Huang Y (2020). Integration of Antiangiogenic Therapy with Cisplatin and Gemcitabine Chemotherapy in Patients with Nasopharyngeal Carcinoma. Clin Cancer Res.

[233] Bhuvaneswari R, Gan YY, Yee KK, Soo KC, Olivo M (2007). Effect of hypericin-mediated photodynamic therapy on the expression of vascular endothelial growth factor in human nasopharyngeal carcinoma. Int J Mol Med.

[234] Sun Q, Wang Y, Ji H, Sun X, Xie S, Chen L (2022). Lenvatinib for effectively treating antiangiogenic drug-resistant nasopharyngeal carcinoma. Cell Death Dis.

[235] He Q, Ren X, Chen J, Li Y, Tang X, Wen X (2016). miR-16 targets fibroblast growth factor 2 to inhibit NPC cell proliferation and invasion via PI3K/AKT and MAPK signaling pathways. Oncotarget.

[236] Carbone C, Tamburrino A, Piro G, Boschi F, Cataldo I, Zanotto M (2016). Combined inhibition of IL1, CXCR1/2, and TGFβ signaling pathways modulates in-vivo resistance to anti-VEGF treatment. Anticancer Drugs.

[237] Shergold AL, Millar R, Nibbs RJB (2019). Understanding and overcoming the resistance of cancer to PD-1/PD-L1 blockade. Pharmacol Res.

[238] Wang H, Wang W, Fan S (2022). Emerging roles of lncRNA in Nasopharyngeal Carcinoma and therapeutic opportunities. Int J Biol Sci.

[239] Xiao Y, Qing J, Li B, Chen L, Nong S, Yang W (2020). TIM-3 Participates in the Invasion and Metastasis of Nasopharyngeal Carcinoma via SMAD7/SMAD2/SNAIL1 Axis-Mediated Epithelial-Mesenchymal Transition. Onco Targets Ther.

[240] Yang R, Sun L, Li CF, Wang YH, Yao J, Li H (2021). Galectin-9 interacts with PD-1 and TIM-3 to regulate T cell death and is a target for cancer immunotherapy. Nat Commun.

[241] Pickup M, Novitskiy S, Moses HL (2013). The roles of TGFβ in the tumour microenvironment. Nat Rev Cancer.

[242] Wu Q, Tian AL, Li B, Leduc M, Forveille S, Hamley P, et al. IGF1 receptor inhibition amplifies the effects of cancer drugs by autophagy and immune-dependent mechanisms. J Immunother Cancer. 2021;9(6):e002722. 10.1136/jitc-2021-002722 PMC8204183 34127545

[243] Ma XM, Luo YF, Zeng FF, Su C, Liu X, Li XP (2022). TGF-β1-Mediated PD-L1 Glycosylation Contributes to Immune Escape via c-Jun/STT3A Pathway in Nasopharyngeal Carcinoma. Front Oncol.

[244] Herbst RS, Soria JC, Kowanetz M, Fine GD, Hamid O, Gordon MS (2014). Predictive correlates of response to the anti-PD-L1 antibody MPDL3280A in cancer patients. Nature.

[245] Chen L, Han X (2015). Anti-PD-1/PD-L1 therapy of human cancer: past, present, and future. J Clin Investig.

[246] Zhou J, Shi Y-H, Liu B, Jia W-D, Gu S, Qin Y, et al. A phase Ib, multicenter, open-label study to assess the safety, tolerability, and preliminary efficacy of sintilimab plus IBI310 (anti-CTLA4 mAb) in patients with advanced hepatocellular carcinoma. J Clin Oncol. 2022;40(4_suppl):421.

[247] Lian B, Cui C, Si L, Sheng X, Chi Z, Mao L, et al. IBI310 monotherapy or in combination with sintilimab in patients with advanced melanoma: An open-label phase Ia/1b study. J Clin Oncol. 2020;38(15_suppl):e15111-e.

[248] Van den bossche V, Zaryouh H, Vara-Messler M, Vignau J, Machiels J-P, Wouters A, et al. Microenvironment-driven intratumoral heterogeneity in head and neck cancers: clinical challenges and opportunities for precision medicine. Drug Resist Updat. 2022;60:100806. 10.1016/j.drup.2022.100806 35121337

[249] Sellam LS, Zappasodi R, Chettibi F, Djennaoui D, Yahi-Ait Mesbah N, Amir-Tidadini ZC (2020). Silibinin down-regulates PD-L1 expression in nasopharyngeal carcinoma by interfering with tumor cell glycolytic metabolism. Arch Biochem Biophys.

[250] Chia WK, Teo M, Wang WW, Lee B, Ang SF, Tai WM (2014). Adoptive T-cell transfer and chemotherapy in the first-line treatment of metastatic and/or locally recurrent nasopharyngeal carcinoma. Mol Ther.

[251] Huang J, Fogg M, Wirth LJ, Daley H, Ritz J, Posner MR (2017). Epstein-Barr virus-specific adoptive immunotherapy for recurrent, metastatic nasopharyngeal carcinoma. Cancer.

